# Exploring longitudinal relationships among Alzheimer's disease biomarkers

**DOI:** 10.1002/alz.71711

**Published:** 2026-07-31

**Authors:** Benjamin Saef, Kellen K. Petersen, Katherine Volluz, Yan Li, Duygu Tosun, Marta Mila‐Aloma, Leslie M. Shaw, Henrik Zetterberg, Jeffrey L. Dage, Carrie E. Rubel, Kyle Ferber, Lei Du‐Cuny, Janaky Coomaraswamy, Michael Baratta, Yulia Mordashova, Ziad S. Saad, Gallen Triana‐Baltzer, Nicholas J. Ashton, Emily A. Meyers, Erin G. Rosenbaugh, J. Martin Sabandal, Anthony W. Bannon, William Z. Potter, Alzheimer's Disease Neuroimaging Initiative (ADNI), Foundation for the National Institutes of Health (FNIH) Biomarkers Consortium Plasma Aβ and Phosphorylated Tau as Predictors of Amyloid and Tau Positivity in Alzheimer's Disease Project Team, Suzanne E. Schindler

**Affiliations:** ^1^ Department of Neurology Washington University in St. Louis St. Louis Missouri USA; ^2^ Northern California Institute for Research and Education San Francisco California USA; ^3^ Department of Radiology and Biomedical Imaging University of California San Francisco San Francisco California USA; ^4^ Department of Pathology and Laboratory Medicine University of Pennsylvania Philadelphia Pennsylvania USA; ^5^ Institute of Neuroscience and Physiology Department of Psychiatry and Neurochemistry The Sahlgrenska Academy at University of Gothenburg Mölndal Sweden; ^6^ Clinical Neurochemistry Laboratory Sahlgrenska University Hospital Mölndal Sweden; ^7^ UK Dementia Research Institute Fluid Biomarkers Laboratory UK DRI at UCL London UK; ^8^ Department of Neurodegenerative Disease UCL Queen Square Institute of Neurology London UK; ^9^ Hong Kong Center for Neurodegenerative Diseases Clear Water Bay Hong Kong China; ^10^ Wisconsin Alzheimer's Disease Research Center University of Wisconsin School of Medicine and Public Health University of Wisconsin–Madison Madison Wisconsin USA; ^11^ Department of Neurology Indiana University School of Medicine Indianapolis Indiana USA; ^12^ Stark Neurosciences Research Institute Indiana University School of Medicine Indianapolis Indiana USA; ^13^ Biogen Cambridge Massachusetts USA; ^14^ AbbVie Deutschland GmbH & Co. KG Ludwigshafen am Rhein Rheinland‐Pfalz Germany; ^15^ Takeda Pharmaceutical Company Ltd. Cambridge Massachusetts USA; ^16^ Precision Measures Johnson & Johnson San Diego California USA; ^17^ Banner Alzheimer's Institute Phoenix Arizona USA; ^18^ Banner Sun Health Research Institute Sun City Arizona USA; ^19^ Alzheimer's Association Chicago Illinois USA; ^20^ The Foundation for the National Institutes of Health North Bethesda Maryland USA; ^21^ AbbVie North Chicago Illinois USA; ^22^ Highly qualified expert Philadelphia Pennsylvania USA

**Keywords:** Alzheimer's disease, blood biomarkers, data visualization, neuroimaging, plasma biomarkers, R shiny app

## Abstract

**INTRODUCTION:**

While most studies of Alzheimer's disease (AD) examine cross‐sectional relationships among biomarkers, longitudinal relationships are also highly relevant.

**METHODS:**

This study in the Alzheimer's Disease Neuroimaging Initiative cohort (*n* = 373) used non‐parametric Spearman correlations to explore the relationships of baseline values and rates of change in plasma biomarkers and rates of change in key AD outcomes.

**RESULTS:**

Compared to rates of change of plasma biomarkers, baseline values of plasma biomarkers were more strongly associated with rates of change in key AD outcomes. Change in amyloid positron emission tomography (PET) was most strongly associated with baseline values of amyloid beta (Aβ)42/Aβ40 and phosphorylated tau (p‐tau)217, especially in amyloid PET–‐negative individuals. Changes in cortical thickness and measures of cognition were most strongly associated with baseline p‐tau217, especially in amyloid PET–positive individuals.

**DISCUSSION:**

Baseline p‐tau217 is associated with rates of change of amyloid pathology and cognition. Visualization tools were developed to enable researchers to explore AD biomarker relationships.

## Highlights

- Baseline plasma biomarkers were associated with rates of change in Alzheimer's disease (AD) outcomes.
- Associations between rates of change in plasma biomarkers and AD outcomes were weak.
- Novel web‐based applications visualized longitudinal AD biomarker relationships.

## 1 BACKGROUND

Alzheimer's disease (AD) is the most common cause of dementia, and affects > 57 million individuals worldwide.^1^ AD is characterized by the accumulation of extracellular amyloid plaques and intracellular tau tangles, which begins during an asymptomatic phase lasting a decade or longer^2^ and often leads to neurodegeneration and progressive cognitive impairment.^3^ Notably, co‐pathologies may affect the expression of cognitive symptoms associated with amyloid and tau pathology.^4^ However, even in patients with typical AD dementia, relationships between biomarkers and cognition may vary due to numerous factors including age, medical comorbidities, and social determinants of health.^5^ Now that treatments targeting one of the underlying pathologies of AD, amyloid beta (Aβ) plaques, are clinically available,^6^, ^7^ there is increasing interest in understanding the heterogeneity of AD to individualize diagnosis and care. While most AD biomarker studies have examined variables on a group level with cross‐sectional data, studies of individual‐level trajectories may provide insights into the heterogeneity of AD across a group, including the complexity of relationships between various biomarkers and between biomarkers and cognition.^8^

Different aspects of AD can be measured via imaging and cognitive measures. Positron emission tomography (PET) imaging of radioligands that specifically bind amyloid can measure the lifetime accumulation of amyloid plaques, including in asymptomatic individuals^9^. PET imaging of radioligands that bind insoluble tau pathology reveals the levels of neurofibrillary tangles in different brain regions, which are correlated with AD symptoms.^9^, ^10^, ^11^, ^12^ Cortical thickness reflects neurodegeneration due to AD and other pathologies and is associated with dementia symptoms.^13^ The presence and severity of dementia symptoms are quantified with the Clinical Dementia Rating (CDR).^14^ The Alzheimer's Disease Cooperative Study Preclinical Alzheimer Cognitive Composite (ADCS‐PACC) was used to measure early cognitive decline^15^. Together, these multimodal assessments provide a relatively comprehensive view of AD pathology and symptoms.

Compared to imaging modalities or cerebrospinal fluid tests, blood biomarker (BBM) tests that reflect AD pathology are more accessible, acceptable, lower burden, potentially inexpensive, and scalable^15^. A lower ratio of Aβ peptide 42 to 40 (Aβ42/Aβ40) reflects sequestration of Aβ42 into amyloid plaques and changes very early in AD.^16^, ^17^ Tau is phosphorylated at multiple sites in response to amyloid deposition,^18^, ^19^ even before significant neurofibrillary tangles are present, including at positions 181 and 217 (p‐tau181 and p‐tau217).^20^, ^21^, ^22^, ^23^ A biomarker of astrocytic activation, glial fibrillary acidic protein (GFAP), also increases in response to amyloid pathology^24^. Neurofilament light chain (NfL) increases with neuroaxonal damage in a range of neurological conditions.^25^, ^26^ The Foundation for the National Institutes of Health (FNIH) Biomarkers Consortium recently examined the cross‐sectional relationships between key AD outcomes and BBM tests in the Alzheimer's Disease Neuroimaging Initiative (ADNI) cohort^27^. The FNIH dataset, which also includes longitudinal BBM data on these measures, enables examination of the complex relationships among BBMs, imaging measures, and cognitive outcomes^17^.

In the current descriptive study using the FNIH dataset, we first explore non‐parametric correlations between plasma biomarkers and key AD outcomes. We examine the associations of baseline values and rates of change in plasma biomarkers with rates of change in amyloid PET, tau PET, cortical thickness, and dementia symptoms. Next, we introduce novel interactive web‐based applications to visualize individual trajectories in AD biomarker relationships. The raindrop application visualizes relationships between baseline biomarker values and rates of change in other measures. The time trails application visualizes dynamic relationships between rates of change in two biomarkers. These analyses and tools are not designed to test specific hypotheses, but rather to provide a broad view of the relationships between AD biomarkers and to help investigators see biomarker relationships in a new way that improves appreciation of the heterogeneity of biomarker relationships across individuals.

**RESEARCH IN CONTEXT**

1. **Systematic review**: The literature was reviewed using PubMed. Most studies of Alzheimer's disease (AD) biomarkers have focused on cross‐sectional differences between groups rather than longitudinal relationships between biomarkers and AD outcomes. There are currently no open‐source tools designed to visualize longitudinal AD biomarker relationships.
2. **Interpretation**: Non‐parametric correlations were used to provide an overall view of the relationships of baseline values and rates of change in plasma biomarkers with rates of change in amyloid positron emission tomography (PET), tau PET, cortical thickness, and dementia symptoms. Novel interactive web‐based applications were introduced to visualize individual trajectories in AD biomarker relationships.
3. **Future directions**: This article provides new tools to enable researchers to see and explore longitudinal AD biomarker data, facilitating the appreciation of the non‐linearity and heterogeneity of biomarker relationships. The code is shared to facilitate the development and refinement of similar tools, including those using other datasets, to inspire the creation of novel hypotheses and inform analytical approaches.

## 2 METHODS

### 2.1 Participants and apolipoprotein E genotyping

The current analyses examined plasma biomarker data generated by the FNIH Biomarker Consortium using the ADNI cohort (adni.loni.usc.edu). ADNI was launched in 2003 as a public–private partnership led by Principal Investigator Michael W. Weiner, MD. The primary goal of ADNI has been to test whether serial magnetic resonance imaging (MRI), PET, other biological markers, and clinical and neuropsychological assessment can be combined to measure the progression of AD. Written informed consent was obtained from each participant or their legally authorized representative. Race and sex were self‐identified. Apolipoprotein E (*APOE*) genotyping was performed as part of the ADNI protocol.

For correlation analyses, participants were included who had data from the FNIH Biomarker Consortium study that represented two or more plasma samples within 6 years. For the web‐based applications, participants were included who had data from the FNIH Biomarker Consortium study.

### 2.2 Diagnostic and cognitive measure*s*

Participants underwent annual clinical assessments that included a detailed interview of a collateral source and a neurological examination of the participant. The PACC, CDR, and the CDR Sum of Boxes (CDR‐SB) were used to assess the presence of and, if present, the severity of dementia.^14^, ^15^

### 2.3 Plasma biomarker assays

Longitudinal data from the following plasma biomarker tests were examined: C2N Diagnostics’ PrecivityAD2%p‐tau217, p‐tau217, and Aβ42/Aβ40; Fujirebio Diagnostics’ Lumipulse p‐tau217 and Aβ42/Aβ40 (with research use only kits); ALZpath's Quanterix p‐tau217; Janssen's LucentAD Quanterix p‐tau217; Roche Diagnostics’ NeuroToolKit p‐tau181, Aβ42/Aβ40, GFAP, and NfL; and Quanterix's Neurology 4‐Plex p‐tau181, Aβ42/Aβ40, GFAP, and NfL. Technical details on the assays have been published^28^ and are also available in the study methodology report, which can be accessed from the ADNI database.

### 2.4 Amyloid PET, tau PET, and MRI variables

Amyloid PET imaging used florbetapir (18F‐AV‐45) and was conducted across all ADNI sites following standardized protocols^29^. A global standardized uptake value ratio (SUVR) was calculated across a cortical summary region and normalized using either cerebral white matter or a composite reference region, as described previously^18^. The threshold for amyloid positivity was set at 20 Centiloids (CL), consistent with established practice for this dataset^28^. Tau PET imaging with flortaucipir (18F‐AV‐1451) was performed at all ADNI sites^18^. Two distinct meta‐regions of interest (meta‐ROIs) were used to quantify tau deposition: (1) Early‐changing tau PET measure: the mesial‐temporal meta‐ROI, comprising the entorhinal cortex, parahippocampal gyrus, and amygdala. (2) Late‐changing tau PET measure: the temporal‐parietal meta‐ROI, comprising the banks of the superior temporal sulcus; cuneus; inferior and superior parietal cortices; inferior, middle, and superior temporal gyri; isthmus of the cingulate cortex; lateral occipital cortex; lingual gyrus; posterior cingulate; precuneus; and superior marginal gyrus. Positivity thresholds (cut‐offs) for both the early and late tau PET measures were applied as previously defined.^18^

Structural brain MRI data were acquired using a T1‐weighted 3T sequence (either 3D magnetization‐prepared rapid gradient echo or inversion recovery‐prepared fast spoiled gradient recall) with sagittal slices. Cortical parcellation was performed using the Desikan–Killiany–Tourville (DKT) volumetric atlas^18^. A composite meta‐ROI cortical thickness measurement was calculated, including the following regions: entorhinal cortex, fusiform gyrus, parahippocampal gyrus, middle temporal gyrus, inferior temporal gyrus, and angular gyrus.^30^ To ensure data comparability, harmonization procedures were applied to the MRI data.^18^ To account for the effects of normal aging, age‐related variance was estimated in the harmonized meta‐ROI cortical thickness using cognitively unimpaired and amyloid‐negative individuals. This variance was then regressed out from the corresponding measures in all individuals using age and quadratic age as predictors.^18^

### 2.5 Statistical methods

For participant characteristics tables, the significance of differences by amyloid PET status was evaluated with Wilcoxon rank‐sum tests for continuous variables and chi‐squared or Fisher exact tests for categorical variables.

Rates of change were estimated using linear regression of all available data points collected within 6 years after the baseline plasma sample. Spearman correlations were used to evaluate the relationships of baseline values and rates of change in plasma biomarkers with amyloid PET CL, tau PET measures, cortical thickness measures, and dementia severity as measured by CDR‐SB or PACC. Comparisons between Spearman correlations were performed by bootstrapping with 12,500 iterations. All tests were two tailed with significance at *P* = 0.05. All analyses were performed in R using the stats, boot, tidyr, and RVAideMemoire packages. Data from this study are available to researchers upon request at adni.loni.usc.edu.

### 2.6 Generation of the R Shiny apps and related datasets

A dataset was created to enable visualization of dynamic relationships. The first available plasma collection date was set as the baseline (time zero) for all other study dates. For each variable (plasma biomarker, imaging, or cognitive measure), the estimated values for each individual at 10‐day increments between adjacent observed data points were interpolated using a linear regression. The interpolated data for all variables was then merged into a single dataset by time from baseline plasma collection.

The R Shiny and ggplot2 packages were used to create interactive animated plots of biomarker changes over time. The apps ensure installation of required packages including shiny, ggforce, and dplyr. Code for the apps is available at https://github.com/WashUFluidBiomarkers/Dynamic‐Visualization.

## 3 RESULTS

### 3.1 Participants

The cohort included 373 individuals, of whom 178 (47.7%) were female, 125 (33.5%) carried an *APOE* ε4 allele, and 347 (93.0%) self‐identified as White (Table 1). At their baseline plasma sample, the median age was 72.7 years (interquartile range [IQR] 67.8–78.0 years), 141 (37.8%) were amyloid PET positive (CL > 20), and 179 (48.0%) had cognitive impairment (CDR > 0). Most individuals (174, 97.2%) with cognitive impairment at baseline had mild cognitive impairment or very mild dementia (CDR 0.5); only 5 (2.8%) had dementia (CDR ≥ 1). A sub‐cohort of 91 participants had data for tau PET: 24 (26.4%) were tau PET positive in earlier changing brain regions (mesial‐temporal meta‐ROI) and 16 (17.6%) were tau PET positive in later changing brain regions (temporal‐parietal meta‐ROI). A sub‐cohort of 314 participants had brain MRI data and 274 (87.3%) had brain atrophy. Values for baseline plasma biomarkers are shown in Table S1 in supporting information. Compared to those who were amyloid PET negative (CL ≤ 20), amyloid PET positive (CL > 20) individuals had lower plasma Aβ42/Aβ40 and higher plasma p‐tau217, p‐tau181, GFAP, and NfL (*P* < 0.0001 for all assays).

**TABLE 1:** Cohort characteristics.

Characteristic	Full cohort		Amyloid PET negative		Amyloid PET positive		*P* =
	*n* =	Values	*n* =	Values	*n* =	Values	
**Demographics**							
Age (years)	373	72.7 (67.8–78.0)	232	71.3 (66.7–76.7)	141	75.9 (69.7–79.4)	0.0001
Sex (n, % female)	373	178, 47.7%	232	112, 48.3%	141	66, 46.8%	0.80
*APOE* genotype (22/23/24/33/34/44^a^)	373	1/33/6/214/101/18	232	1/28/1/151/44/7	141	0/5/5/63/57/11	<0.0001
*APOE* ε4 carrier status (n, % carrier)	373	125, 33.5%	232	52, 22.4%	141	73, 51.8%	<0.0001
Years of education	373	16 (14–18)	232	17 (15–19)	141	16 (14–18)	0.004
Race (Black/White/other)	373	14/347/12	232	10/212/10	141	4/135/2	0.23
CDR (0/0.5/1+)	373	194/174/5	232	135/97/0	141	59/77/5	0.0004
CDR Sum of Boxes	373	0.5 (0–1)	232	0 (0–1)	141	0.5 (0–1.5)	<0.0001
PACC	373	−1.2 (−4.3 to 1.4)	232	0.0 (–2.3 to 1.9)	141	−3.1 (−8.5 to −0.1)	<0.0001
**Amyloid PET**							
Amyloid PET Centiloid	373	8 (−4 to 49)	232	−1 (−8 to 6)	141	61 (41–92)	<0.0001
Cerebral White Matter	342	0.661 (0.621–0.75)	215	0.634 (0.61–0.661)	127	0.789 (0.722–0.822)	<0.0001
Adjusted summary SUVR							
Composite reference region	373	0.716 (0.673–0.865)	232	0.686 (0.655–0.711)	141	0.929 (0.832–1.01)	<0.0001
Adjusted summary SUVR							
**Tau PET**							
Tau PET, early (positivity)	91	24, 26.4%	52	6, 11.5%	39	18, 46.2%	0.0002
Tau PET SUVR, early	91	1.2 (1.13–1.33)	52	1.16 (1.08–1.24)	39	1.29 (1.18–1.48)	<0.0001
Tau PET, late (positivity)	91	16, 17.6%	52	4, 7.7%	39	12, 30.8%	0.004
Tau PET SUVR, late	91	1.13 (1.07–1.21)	52	1.1 (1.05–1.13)	39	1.19 (1.13–1.31)	<0.0001
**Cortical thickness**							
Brain atrophy (positivity)	314	274, 87.3%	202	191, 94.6%	112	83, 74.1%	<0.0001
Cortical thickness volume	314	2.8 (2.67–2.9)	202	2.82 (2.72–2.91)	112	2.74 (2.55–2.84)	<0.0001

*Note*: Continuous values are presented as the median with the interquartile range. The significance of differences by amyloid PET status were evaluated with Wilcoxon rank‐sum tests for continuous variables and chi‐squared or Fisher exact tests for categorical variables. All tests were two sided and not adjusted for covariates or multiple comparisons.

Abbreviations: 22, ε2/ε2; 23, ε2/ε3; 24, ε2/ε4; 33, ε3/ε3; 34, ε3/ε4; 44, ε4/ε4; *APOE*, apolipoprotein E; CDR, Clinical Dementia Rating; PACC, Preclinical Alzheimer Cognitive Composite; PET, positron emission tomography; SUVR, standardized uptake value ratio.

We first examined associations between plasma biomarkers and rates of change in key AD outcomes. The goal was to provide a broad and descriptive overview of the relationships between many biomarker measures over time. Because many biomarkers have non‐linear trajectories, data collected > 6 years from the baseline plasma sample were excluded to enable a model more closely approximating linearity, which aligns with the visualization strategy for our visualization applications using piece‐wise linear regression. With these criteria, data from two to three plasma samples were typically included and the median time between the baseline and the last plasma biomarker value was ≈ 4.0 years for all analytes (Table S2 in supporting information). For data from imaging and clinical assessments performed within 6 years of the baseline plasma sample, the median time from the baseline to the last imaging or clinical assessment was 4.1 years for amyloid PET (median of 3 scans), 2.0 years for tau PET (median of 2 scans), 4.1 years for brain MRI (median of 4 scans), and 5.1 years for clinical assessment (median of 5 assessments; Table S3 in supporting information).

### 3.2 Correlations between baseline plasma biomarker values and rates of change

The correlations between baseline plasma biomarker values and rates of change in plasma, imaging, and cognitive measures were examined using a consistent approach across all modalities. Rates of change were estimated using linear regression because few points were represented for most measures and the follow‐up time was short (by design, < 6 years; see Tables S2 and S3). Correlations were performed with non‐parametric Spearman correlations because of expected non‐linearity of many relationships. Statistical significance was not adjusted for multiple comparisons given the exploratory and descriptive nature of this study.

**FIGURE 1:**
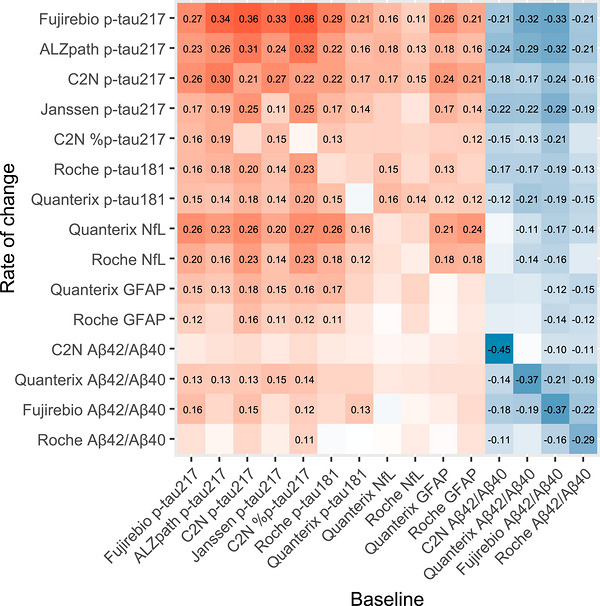
Correlation matrix of baseline plasma biomarker values and plasma biomarker rates of change in the full cohort. The unadjusted Spearman correlation of baseline plasma biomarker values and rates of change are shown for nominally significant correlations (*P* < 0.05, not adjusted for multiple comparisons). Redder colors are used for larger positive correlations and bluer colors are used for larger negative correlations. Aβ, amyloid beta; GFAP, glial fibrillary acidic protein; NfL, neurofilament light chain; p‐tau, phosphorylated tau

The correlations between baseline plasma biomarker values and rates of change in plasma biomarkers in the full cohort are shown in Figure 1. The absolute correlations were greatest for Aβ42/Aβ40 as measured by the same assay (*ρ* = –0.45 for C2N Aβ42/Aβ40, –0.37 for both Quanterix and Fujirebio Aβ42/Aβ40, and –0.29 for Roche Aβ42/Aβ40). For the five p‐tau217 measures, the correlations were generally greatest for Fujirebio p‐tau217 (*ρ* = 0.27 to 0.36). Correlations between baseline values and rates of change were lower for p‐tau181, NfL, and GFAP.

**FIGURE 2:**
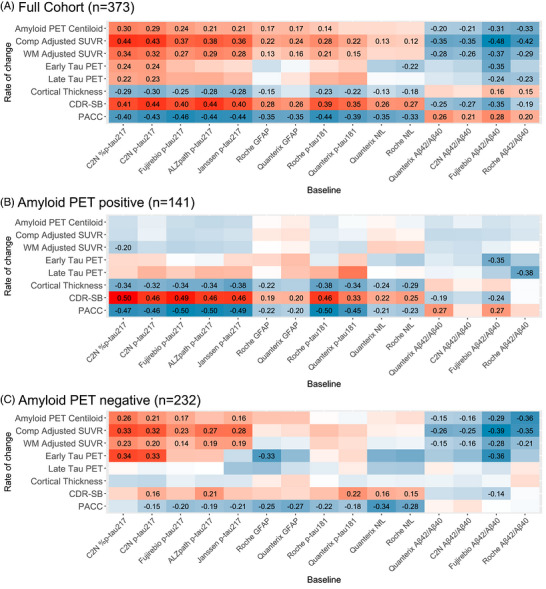
Correlation matrix of baseline plasma biomarker values and rates of change in key AD outcomes. The unadjusted Spearman correlation of baseline plasma biomarker values and rates of change in the outcome are shown for nominally significant correlations (*P* < 0.05, not adjusted for multiple comparisons). Redder colors are used for larger positive correlations and bluer colors are used for larger negative correlations. Aβ, amyloid beta; AD, Alzheimer's disease; CDR‐SB, Clinical Dementia Rating Sum of Boxes; GFAP, glial fibrillary acidic protein; NfL, neurofilament light chain; PACC, Preclinical Alzheimer Cognitive Composite; PET, positron emission tomography; p‐tau, phosphorylated tau; SUVR, standardized uptake value ratio; WM, white matter

Using a similar approach, baseline plasma biomarker values were correlated with rates of change in imaging and cognitive measures (Figure 2). Rates of change in amyloid PET CL, white matter adjusted amyloid PET SUVR, and composite reference adjusted amyloid PET SUVR were most strongly correlated with baseline plasma Aβ42/Aβ40 measures (Tables S4–S6 in supporting information). The highest correlations for amyloid PET CL were with baseline Roche Aβ42/Aβ40 (ρ = –0.329 [95% confidence interval (95% CI) −0.232 to −0.422]) and Fujirebio Lumipulse Aβ42/Aβ40 (ρ = –0.310 [95% CI –0.209 to –0.403]). Absolute correlations for rates of change in amyloid PET CL and baseline plasma Aβ42/Aβ40 measures were lower in the amyloid PET–positive sub‐cohort (*ρ* = −0.051 to −0.114), and higher in the amyloid PET–negative sub‐cohort (ρ = –0.150 to –0.359). The rates of change in tau PET in both the early changing (mesial‐temporal meta‐ROI) and late (temporal‐parietal meta‐ROI) changing brain regions were most strongly correlated with baseline plasma Aβ42/Aβ40 and p‐tau217 measures (Tables S7 and S8 in supporting information). The highest absolute correlations of early tau PET were with baseline Fujirebio Lumipulse Aβ42/Aβ40 (*ρ* = −0.348 [95% CI −0.155 to −0.513]). Correlations with most baseline plasma biomarkers were lower in the amyloid PET–positive sub‐cohort compared to the amyloid PET–negative sub‐cohort.

Rates of change in cortical thickness were most strongly correlated with baseline plasma p‐tau217 measures (Table S9 in supporting information). Correlations were similar across assays and ranged from ρ = –0.253 to –0.301 in the full cohort. Notably, correlations were higher in the amyloid PET–positive sub‐cohort (ρ = –0.318 to –0.384) and lower in the amyloid PET–negative sub‐cohort (ρ = –0.044 to –0.124). There was a similar pattern for correlations between rates of change in cognitive measures and p‐tau217 (Table S10 and S11 in supporting information). For the CDR‐SB, correlations with p‐tau217 were similar across assays and ranged from ρ = 0.397 to 0.437 in the full cohort, were higher in the amyloid PET–positive sub‐cohort (ρ = 0.457 to 0.504), and were lower in the amyloid PET–negative sub‐cohort (ρ = 0.081 to 0.205). For the PACC, correlations with p‐tau217 ranged from ρ = –0.399 to –0.458 in the full cohort, were highest in the amyloid PET–positive sub‐cohort (ρ = –0.460 to –0.502), and were lower in the amyloid PET–negative sub‐cohort (ρ = –0.075 to –0.207).

### 3.3 Correlations between rates of change in plasma biomarkers and rates of change in other measures

**FIGURE 3:**
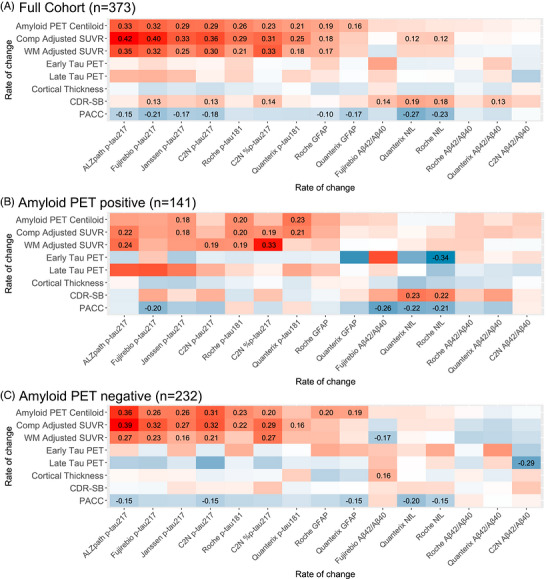
Correlation matrix of rates of change plasma biomarkers and rates of change in key AD outcomes. The unadjusted Spearman correlation of rates of change in plasma biomarkers and rates of change in the outcome are shown for nominally significant correlations (*P* < 0.05, not adjusted for multiple comparisons). Redder colors are used for larger positive correlations and bluer colors are used for larger negative correlations. Aβ, amyloid beta; AD, Alzheimer's disease; CDR‐SB, Clinical Dementia Rating Sum of Boxes; GFAP, glial fibrillary acidic protein; NfL, neurofilament light chain; PACC, Preclinical Alzheimer Cognitive Composite; PET, positron emission tomography; p‐tau, phosphorylated tau; SUVR, standardized uptake value ratio; WM, white matter

Next, rates of change in plasma biomarkers were correlated with rates of change in imaging and cognitive measures (Figure  3). These correlations were generally very low. The rate of change in amyloid PET CL, white matter adjusted amyloid PET SUVR, and composite reference adjusted amyloid PET SUVR, were consistently correlated with the rates of change in plasma p‐tau217, p‐tau181, and GFAP in the full cohort (Tables S12–S14 in supporting information). Correlations between rates of change in tau PET (early or late) or cortical thickness (Tables S15–S17 in supporting information) and rates of change in plasma biomarkers were very low. The rates of change in the CDR‐SB and PACC were very weakly associated with the rates of change in plasma NfL (ρ = 0.177 to 0.191 and ρ = –0.230 to –0.271, respectively), which were slightly higher in the amyloid PET–positive sub‐cohort (ρ = 0.217 to 0.230 and ρ = –0.214 to –0.222) and lower in the amyloid PET–negative sub‐cohort (ρ = 0.072 to 0.074 and ρ = –0.149 to –0.198; Tables S18 and S19 in supporting information).

### 3.4 Dynamic visualization applications

Time series were created to enable visualization of dynamic relationships between biomarkers across the entire period of follow‐up (i.e., were not truncated at 6 years as was done for correlations). For each individual, the date at baseline plasma collection was set as time zero and the estimated values for each biomarker or cognitive measure at intermediate time points (every 10 days) were interpolated by linear regression of adjacent measurements. For example, if an individual had a plasma p‐tau217 value of 1 pg/ml at baseline, 2 pg/ml at year 2, and 4 pg/ml at year 4, timepoints between baseline and year 2 were estimated as 0.5 pg/ml x time since baseline + 1 pg/ml; timepoints between year 2 and year 4 were estimated as 1 pg/ml x time since year 2 + 2 pg/ml. Therefore, the time series were not modeled with a single equation that might smooth variation across measurements, but rather used the “raw” measurement values with piece‐wise linear regression of adjacent measurements. The time series for all variables from all individuals were then merged into a single dataset for the entire cohort by time from the baseline plasma collection.

**FIGURE 4:**
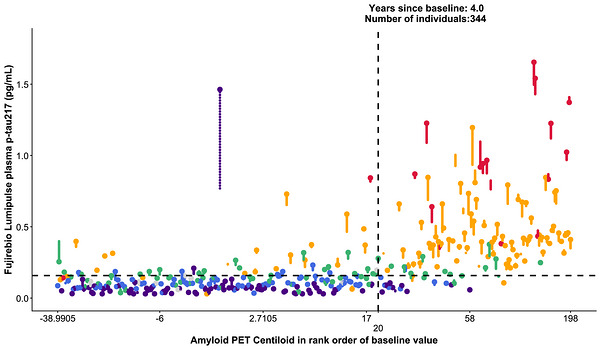
Raindrop dynamic plot of longitudinal plasma Fujirebio Lumipulse p‐tau217 as a function of baseline rank for amyloid PET Centiloid. A snapshot of the dynamic plot is shown for 4 years after plasma collection, when 344 individuals have longitudinal data. Raindrop points (circles) represent the point estimate for the p‐tau217 value for an individual and the associated trail represents the previous 1 year of estimated values (a longer trail signifies faster change between two measurements). Raindrop colors are based on baseline p‐tau217 values. The vertical dashed line represents an amyloid PET Centiloid of 20 and the horizontal dashed line represents a p‐tau217 value of 0.158 pg/ml. PET, positron emission tomography; p‐tau, phosphorylated tau

The first type of dynamic plot generated using this dataset is termed a “Raindrop plot,” as individual trajectories extend vertically up or down (Figure 4, Video 1). To avoid overplotting and facilitate visualization of individual trajectories, the baseline rank of the measure is plotted on the *x* axis. For each individual, the estimated longitudinal value of the measure at a timepoint is plotted on the *y* axis with a circle (the raindrop). A line extends from the raindrop to demonstrate the estimated values over the previous year, with longer lines demonstrating a faster rate of change between two measurements. Large differences between adjacent measurements appear as a long, dashed line, but may reflect measurement error rather than biological change (for example, see long purple line in the upper left‐hand quadrant of Figure 4). A web‐based application enables users to select which biomarkers are presented on each axis, adjust the time since baseline plasma collection, color the raindrops by baseline or longitudinal biomarker values, adjust reference lines, and output a customized video (https://amyloid.shinyapps.io/ADNI_Raindrop_Plots/).

**FIGURE 5:**
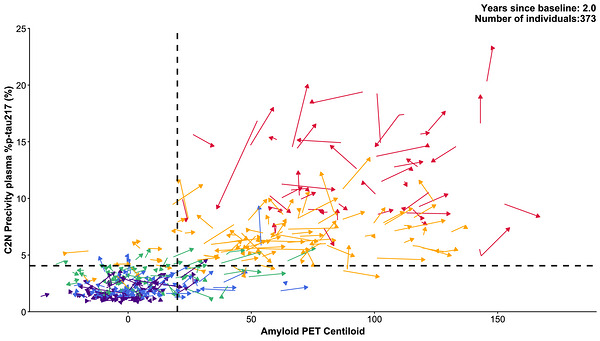
Time trails plot of longitudinal plasma C2N Precivity %p‐tau217 as a function of amyloid PET Centiloid. A snapshot of the dynamic plot is shown at 2 years after plasma collection, when 373 individuals have longitudinal data. Arrows represent the point estimate for the %p‐tau217 value for an individual and the associated trail represents the previous 1 year of estimated values (a longer trail signifies faster change between two measurements). Trail colors are based on baseline %p‐tau217 values. The vertical dashed line represents an amyloid PET Centiloid of 20 and the horizontal dashed line represents a %p‐tau217 value of 4.06%. PET, positron emission tomography; p‐tau, phosphorylated tau

The second type of dynamic plot, which uses the same dataset, is termed a “Time trails plot,” as it shows the relationships between two variables over time (Figure 5, Video 2). For each individual, the estimated longitudinal value of two measures at a timepoint is plotted on the *x* axis and *y* axis with an arrow. A line extends from the arrow to demonstrate the estimated values over the previous year, with longer lines demonstrating a faster rate of change. A web‐based application with features similar to the raindrop app allows users to visualize the dynamic plots and output a customized video (https://amyloid.shinyapps.io/ADNI_Time_Trails/).

## 4 DISCUSSION

Our previous work examined the cross‐sectional relationships of plasma biomarkers with key AD outcomes: amyloid PET, tau PET, cortical thickness, and cognitive impairment.^28^ In the current study, we explored the relationships of baseline values and rates of change in plasma biomarkers and rates of change in key AD outcomes. Additionally, we introduced novel web‐based applications to visualize dynamic changes in AD biomarker relationships.

We found that rates of change in both amyloid PET CL and tau PET were most strongly correlated with baseline plasma Aβ42/Aβ40 and p‐tau217 measures, but these correlations were lower in amyloid PET–positive individuals. This suggests that plasma Aβ42/Aβ40 and p‐tau217 track best with amyloid and tau pathology during the early phase of the disease, when amyloid is still accumulating and is below the threshold for positivity^31^, ^32^. Overall, our findings are consistent with other reports that plasma Aβ42/Aβ40 is a very early biomarker of amyloid pathology^18^, ^31^, ^33^, ^34^. As clinical trials work to treat AD earlier^35^, measurement of plasma Aβ42/Aβ40 with highly precise and robust assays may be informative despite the superior performance of p‐tau217 in detecting amyloid and tau pathology in symptomatic individuals^28^.

Rates of change in cortical thickness and dementia severity were most strongly correlated with p‐tau217, and correlations were higher in amyloid PET–positive individuals. These relationships suggest that p‐tau217 not only reflects amyloid and tau pathology^36^, ^37^, but also continues to increase with disease progression and is associated with neurodegeneration and cognitive impairment, highlighting its potential to stage individuals with both preclinical and early symptomatic AD. Our findings were similar to other recent studies with the ADNI dataset, which found that plasma p‐tau217 tracked with disease progression and cognitive changes, and may be useful for monitoring individuals longitudinally^38^, ^39^, ^40^, ^41^, ^42^, ^43^. Although many of these studies focused on developing cut‐offs for screening and risk stratification, we compared the relationship between baseline values and rates of change of plasma biomarkers with rates of change in imaging and cognitive outcomes.

Importantly, rates of change in plasma biomarkers were only weakly correlated with rates of change in key AD outcomes. This is potentially because plasma biomarkers change slowly and differences over short periods of time may be more reflective of measurement error or intra‐individual variability (noise). Minimizing pre‐analytical differences and limiting intra‐ and inter‐assay variability are critically important when analyzing BBMs longitudinally^44^, ^45^, ^46^. Biological mechanisms that affect plasma biomarker levels may also increase noise. Aβ42 and Aβ40 are particularly sensitive to these effects, as peripherally produced Aβ peptides are not associated with amyloid plaque pathology and may obscure brain‐related changes^45^. Likewise, reduced kidney function and certain medications have been shown to affect plasma biomarker levels^47^, ^48^, ^49^, ^50^. Plasma biomarkers with a greater magnitude of change, such as p‐tau217, may provide a higher signal‐to‐noise ratio^51^. Longer follow‐up may also increase the signal‐to‐noise ratio. For now, imaging and cognitive measures may still be required by clinical trials for longitudinal monitoring of AD pathology and progression until plasma biomarkers are developed and validated that track more precisely with these key outcomes.^47^

A major novel contribution of this study is the interactive R Shiny applications. The dynamic visualization of biomarker data with the interactive Raindrops and Time Trails applications allows investigators to view and explore the complex relationships between AD biomarkers. These visualizations demonstrate the non‐linearity and heterogeneity of biomarker relationships across individuals and may inspire the creation of novel hypotheses or inform analytical approaches. The code to create the dynamic visualization applications is shared, facilitating development of similar applications to visualize longitudinal trajectories using other datasets. These applications could also be applied to other types of datasets to enable visualization of longitudinal change.

Limitations of this work include that only a fraction of participants (24.3%) had longitudinal tau PET data, limiting the power to see associations between plasma biomarkers and tau PET. Additionally, to provide a consistent analytical approach to explore an array of diverse variables over a shorter period of follow‐up, the rate of change in a variable for an individual was estimated with linear models and then the rate of change was associated with a second variable via non‐parametric Spearman correlations. The short follow‐up period and small number of longitudinal assessments decreased the power to see differences. While this approach enabled a broad overview, more powerful parametric methods could be implemented to rigorously evaluate associations to test specific hypotheses. Notably, other analyses are being performed with this dataset that use alternative approaches to studying specific longitudinal associations.

Future studies are needed to further understand how plasma biomarker values change in response to non‐AD–related biological processes and whether these biomarkers are affected by demographic factors. Using larger and more diverse cohorts may help us to better understand the heterogeneity of longitudinal trajectories in AD.

## AUTHOR CONTRIBUTIONS

**Alzheimer's Disease Neuroimaging initiative**

## CONFLICT OF INTEREST STATEMENT

K.K.P. has served as a consultant for Eli Lilly. Y.L. is the co‐inventor of the technology “Novel Tau isoforms to predict onset of symptoms and dementia in Alzheimer's disease,” which is in the process of licensing by C2N. L.M.S. receives funding from the NIA for ADNI4 and from NIA for the University of Pennsylvania ADRC P30 for the Biomarker Core and has served on scientific advisory boards and/or as a consultant for Biogen and Roche. H.Z. has served on scientific advisory boards and/or as a consultant for Abbvie, Acumen, Alector, Alzinova, ALZpath, Amylyx, Annexon, Apellis, Artery Therapeutics, AZTherapies, Cognito Therapeutics, CogRx, Denali, Eisai, Enigma, LabCorp, Merck Sharp & Dohme, Merry Life, Nervgen, Novo Nordisk, Optoceutics, Passage Bio, Pinteon Therapeutics, Prothena, Quanterix, Red Abbey Labs, reMYND, Roche, Samumed, ScandiBio Therapeutics AB, Siemens Healthineers, Triplet Therapeutics, and Wave; has given lectures sponsored by Alzecure, BioArctic, Biogen, Cellectricon, Fujirebio, LabCorp, Lilly, Novo Nordisk, Oy Medix Biochemica AB, Roche, and WebMD; is a co‐founder of Brain Biomarker Solutions in Gothenburg AB (BBS), which is a part of the GU Ventures Incubator Program; and is a shareholder of CERimmune Therapeutics (outside submitted work). J.L.D. is an inventor on patents or patent applications of Eli Lilly and Company relating to the assays, methods, reagents and/or compositions of matter for p‐tau assays and Aβ targeting therapeutics. J.L.D. has/is served/serving as a consultant or on advisory boards for Eisai, Abbvie, Genotix Biotechnologies Inc., Gates Ventures, Gate Neurosciences, Dolby Family Ventures, Karuna Therapeutics, AlzPath Inc., Cognito Therapeutics, Inc., Prevail Therapeutics, Neurogen Biomarking, Spear Bio, University of Kentucky, Rush University, Tymora, and Quanterix. J.L.D. has received research support from ADx Neurosciences, Fujirebio, Roche Diagnostics, and Eli Lilly and Company in the past 2 years. J.L.D. has received speaker fees from Eli Lilly and Company and LabCorp. J.L.D. is a founder and advisor for Monument Biosciences. J.L.D. has stock or stock options in Eli Lilly and Company, Genotix Biotechnologies, AlzPath Inc., Neurogen Biomarking, and Monument Biosciences. C.E.R. is an employee of and may own stock in Biogen. K.F. is an employee of and may own stock in Biogen. L.D.‐C. is employed by AbbVie Deutschland GmbH & Co. J.C. receives salary and company stock as compensation for employment with Takeda Pharmaceutical Company Limited. M.B. receives salary and company stock as compensation for employment with Takeda Pharmaceutical Company Limited. Y.M. is employed by AbbVie Deutschland GmbH & Co. Z.S.S. is employed by Johnson & Johnson Innovative Medicine and may receive salary and stock for their employment. G.T.‐B. is employed by Johnson & Johnson Innovative Medicine and may receive salary and stock for their employment. N.J.A has received speaking fees from Eli Lilly, Biogen, Quanterix, and Alamar Biosciences. N.J.A serves on the advisory board for Biogen, Bristol Myers Squibb, New Amsterdam, Janssen, Roche, and TauRx. N.J.A has received consulting fees from Abbvie, Athria, ImaginationLand LLC, MapLight Therapeutics, SpearBio, Neurogen Biomarking, Quanterix, TauRx, Eli‐Lilly, Roche Dx, Beckman Coulter, Janssen, Bristol Myers Squibb, and ImmunoBrain. E.A.M. is employed by the Alzheimer's Association. A.W.B. receives salary and company stock as compensation for his employment with AbbVie Inc. W.Z.P. was previously employed by the National Institute of Mental Health, and he is a stockholder in Merck & Co., Inc. W.Z.P. is a co‐chair emeritus for the FNIH Biomarkers Consortium Neuroscience Steering Committee. W.Z.P. serves as a consultant for Karuna, Neurocrine, Neumarker, Vaaji, and receives grant support from the NIA along with stock options from Praxis Bioresearch. S.E.S. has not received any financial compensation from pharmaceutical or diagnostics companies for > 18 months, but prior to 18 months ago she received honoraria for serving on scientific advisory boards on biomarker testing and education for Eisai and Novo Nordisk, and she received speaking fees from Eisai, Eli Lilly, and Novo Nordisk. She has received honoraria for educational presentations from Medscape, PeerView, and the Academy for Continued Healthcare Learning. She has provided unpaid scientific advising to Acumen, Biogen, Cognito Therapeutics, Danaher, Eisai, Eli Lilly, and Johnson and Johnson Innovative Medicine. B.S., K.E.V., D.T., M.M.‐A., E.G.R., and J.M.S. have no disclosures to report. Author disclosures are available in the Supporting Information.

## CONSENT STATEMENT

Written informed consent was obtained from each participant or their legally authorized representative.

## Supporting information



Supporting Information

Supporting Information

Supporting Information

Supporting Information
