# Supplementary material for: Exploring longitudinal relationships among Alzheimer's disease biomarkers
Source: Alzheimers Dement. 2026 Jul 31;22(8):e71711. doi: 10.1002/alz.71711 (PMC13425622; doi:10.1002/alz.71711)
Supplement: Supplementary file 1 — Supporting Information [file ALZ-22-e71711-s002.docx]

**Supplementary Information**

**Exploring longitudinal relationships**

**between Alzheimer’s disease biomarkers**

**by Saef et al.**

Supplementary Table 1. Baseline plasma biomarker measures stratified by amyloid PET status.

Supplementary Table 2. Plasma biomarker follow-up time stratified by amyloid PET status.

Supplementary Table 3. Imaging and clinical follow-up time stratified by amyloid PET status.

Supplementary Table 4. Correlations between baseline plasma biomarker levels and rates of change in amyloid PET Centiloid.

Supplementary Table 5. Correlations between rates of change plasma biomarker levels and baseline biomarker levels with white matter adjusted amyloid PET SUVR.

Supplementary Table 6. Correlations between rates of change plasma biomarker levels and baseline biomarker levels with composite reference adjusted amyloid PET SUVR.

Supplementary Table 7. Correlations between baseline plasma biomarker levels and rates of change in early tau PET.

Supplementary Table 8. Correlations between baseline plasma biomarker levels and rates of change in late tau PET.

Supplementary Table 9. Correlations between baseline plasma biomarker levels and rates of change in cortical thickness.

Supplementary Table 10. Correlations between baseline plasma biomarker levels and rates of change in CDR-SB.

Supplementary Table 11. Correlations between rates of change plasma biomarker levels and baseline PACC.

Supplementary Table 12. Correlations between rates of change plasma biomarker levels and rates of change in amyloid PET Centiloid.

Supplementary Table 13. Correlations between rates of change plasma biomarker levels and rates of change in white matter adjusted amyloid PET SUVR.

Supplementary Table 14. Correlations between rates of change plasma biomarker levels and rates of change in composite reference adjusted amyloid PET SUVR.

Supplementary Table 15. Correlations between rates of change plasma biomarker levels and rates of change in early tau PET.

Supplementary Table 16. Correlations between rates of change plasma biomarker levels and rates of change in late tau PET.

Supplementary Table 17. Correlations between rates of change plasma biomarker levels and rates of change in cortical thickness.

Supplementary Table 18. Correlations between rates of change plasma biomarker levels and rates of change in CDR-SB.

Supplementary Table 19. Correlations between rates of change plasma biomarker levels and rates of change in PACC.

**Supplementary Table 1. Baseline plasma biomarker measures stratified by amyloid PET status.** Values are presented as the median with the interquartile range. The significance of differences by amyloid PET status were evaluated with Wilcoxon’s rank-sum tests. All tests were two sided and not adjusted for covariates or multiple comparisons.

| **Plasma  Biomarker** | **Full Cohort** | | **Amyloid PET Negative** | | **Amyloid PET Positive** | |  |
| --- | --- | --- | --- | --- | --- | --- | --- |
|  | **n=** | **Values** | **n=** | **Values** | **n=** | **Values** | **p=** |
| **C2N PrecivityAD2** | | | | | | | |
| Aβ42/Aβ40 | 373 | 0.0925 (0.085-0.101) | 232 | 0.0963 (0.090-0.104) | 141 | 0.0861 (0.0814-0.0919) | <0.0001 |
| p-tau217 (pg/ml) | 373 | 1.46 (0.65-2.87) | 232 | 0.65 (0.65-1.51) | 141 | 3.34 (2.34-5.31) | <0.0001 |
| % p-tau217 | 372 | 2.78 (1.54-5.71) | 231 | 1.68 (1.38-2.86) | 141 | 6.41 (4.72-8.95) | <0.0001 |
| **Roche NeuroToolKit** | | | | | | | |
| Aβ42/Aβ40 | 370 | 0.122 (0.113-0.135) | 231 | 0.130 (0.119-0.14) | 139 | 0.114 (0.106-0.121) | <0.0001 |
| p-tau181 (pg/ml) | 370 | 0.941 (0.721-1.3) | 230 | 0.805 (0.664-0.999) | 140 | 1.30 (1.0-1.63) | <0.0001 |
| GFAP (pg/ml) | 371 | 0.0919 (0.0663-0.125) | 231 | 0.0761 (0.0591-0.103) | 140 | 0.123 (0.088-0.168) | <0.0001 |
| NfL (pg/ml) | 371 | 3.31 (2.65-4.48) | 231 | 3.02 (2.47-3.95) | 140 | 3.88 (3.02-5.09) | <0.0001 |
| **Fujirebio Lumipulse** | | | | | | | |
| Aβ42/Aβ40 | 373 | 0.0891 (0.0803-0.097) | 232 | 0.0941 (0.0874-0.10) | 141 | 0.0805 (0.0772-0.087) | <0.0001 |
| p-tau217 (pg/ml) | 373 | 0.108 (0.067-0.243) | 232 | 0.084 (0.052-0.114) | 141 | 0.258 (0.16-0.388) | <0.0001 |
| **ALZpath Quanterix** | | | | | | | |
| p-tau217 (pg/ml) | 373 | 0.298 (0.205-0.558) | 232 | 0.24 (0.183-0.308) | 141 | 0.648 (0.408-0.867) | <0.0001 |
| **Janssen LucentAD Quanterix** | | | | | | | |
| p-tau217 (pg/ml) | 373 | 0.046 (0.030-0.076) | 232 | 0.037 (0.027-0.049) | 141 | 0.082 (0.053-0.129) | <0.0001 |
| **Quanterix Neurology 4-Plex** | | | | | | | |
| Aβ42/Aβ40 | 323 | 0.0609 (0.0538-0.0689) | 209 | 0.0648 (0.058-0.0712) | 114 | 0.0553 (0.0474-0.0602) | <0.0001 |
| p-tau181 (pg/ml) | 323 | 17.8 (12.6-24.7) | 209 | 16 (11.2-20.1) | 114 | 22.9 (17.3-31.9) | <0.0001 |
| GFAP (pg/ml) | 323 | 128 (90.1-180) | 209 | 112 (81.3-148) | 114 | 167 (117-238) | <0.0001 |
| NfL (pg/ml) | 323 | 17.8 (13.5-24.1) | 209 | 16.4 (12.5-22.2) | 114 | 20.3 (15.1-27.1) | <0.0001 |

**Supplementary Table 2. Plasma biomarker follow-up time stratified by amyloid PET status.** The time from the first to the last plasma biomarker data is presented as the median with the interquartile range in number of years. The number of individuals with longitudinal data (n) and number of observations (o) is represented. The significance of differences by amyloid PET status were evaluated with Wilcoxon’s rank-sum tests. All tests were two sided and not adjusted for covariates or multiple comparisons.

| **Characteristic** | **Full Cohort** | | | **Amyloid PET Negative** | | | **Amyloid PET Positive** | | |  |
| --- | --- | --- | --- | --- | --- | --- | --- | --- | --- | --- |
|  | **n=** | **o=** | **Follow-up**  **(years)** | **n=** | **o=** | **Follow-up**  **(years)** | **n=** | **o=** | **Follow-up**  **(years)** | **p=** |
| **C2N Precivity AD2** | | | | | | | | | | |
| Aβ42/Aβ40 | 373 | 3 (2-3) | 4.0 (3.9-4.4) | 232 | 3 (2-3) | 4.0 (3.9-4.4) | 141 | 3 (2-3) | 4.0 (3.6-4.4) | 0.50 |
| %p-tau217 | 373 | 3 (2-3) | 4.0 (3.9-4.4) | 232 | 3 (2-3) | 4.0 (3.9-4.4) | 141 | 3 (3-3) | 4.0 (3.6-4.4) | 0.60 |
| **Roche NeuroToolKi** | | | | | | | | | | |
| Aβ42/Aβ40 | 370 | 3 (2-3) | 4.0 (3.8-4.4) | 231 | 3 (2-3) | 4.0 (3.9-4.4) | 139 | 3 (2-3) | 4.0 (3.6-4.4) | 0.60 |
| p-tau181 (pg/ml) | 370 | 3 (2-3) | 4.0 (3.8-4.4) | 230 | 3 (2-3) | 4.0 (3.9-4.4) | 140 | 3 (2-3) | 4.0 (3.6-4.4) | 0.70 |
| GFAP (pg/ml) | 371 | 3 (2-3) | 4.0 (3.8-4.4) | 231 | 3 (2-3) | 4.0 (3.9-4.4) | 140 | 3 (2-3) | 4.0 (3.6-4.4) | 0.50 |
| NfL (pg/ml) | 371 | 3 (2-3) | 4.0 (3.9-4.4) | 231 | 3 (2-3) | 4.0 (3.9-4.4) | 140 | 3 (2-3) | 4.0 (3.6-4.4) | 0.60 |
| **Fujirebio Lumipulse** | | | | | | | | | | |
| Aβ42/Aβ40 | 373 | 3 (2-3) | 4.0 (3.9-4.4) | 232 | 3 (2-3) | 4.0 (3.9-4.4) | 141 | 3 (2-3) | 4.0 (3.6-4.4) | 0.60 |
| p-tau217 (pg/ml) | 373 | 3 (2-3) | 4.0 (3.9-4.4) | 232 | 3 (2-3) | 4.0 (3.9-4.4) | 141 | 3 (3-3) | 4.0 (3.6-4.4) | 0.60 |
| **ALZpath Quanterix** | | | | | | | | | | |
| p-tau217 (pg/ml) | 373 | 3 (2-3) | 4.0 (3.9-4.4) | 232 | 3 (2-3) | 4.0 (3.9-4.4) | 141 | 3 (2-3) | 4.0 (3.6-4.4) | 0.50 |
| **Janssen LucentAD Quanterix** | | | | | | | | | | |
| p-tau217 (pg/ml) | 373 | 3 (2-3) | 4.0 (3.9-4.4) | 232 | 3 (2-3) | 4.0 (3.9-4.4) | 141 | 3 (3-3) | 4.0 (3.6-4.4) | 0.60 |
| **Quanterix Neurology 4-Plex** | | | | | | | | | | |
| Aβ42/Aβ40 | 323 | 3 (2-3) | 4.0 (3.9-4.5) | 209 | 3 (2-3) | 4.0 (3.8-4.5) | 114 | 3 (2-3) | 4.0 (3.9-4.5) | 0.70 |
| p-tau181 (pg/ml) | 323 | 3 (2-3) | 4.0 (3.9-4.5) | 209 | 3 (2-3) | 4.0 (3.8-4.5) | 114 | 3 (2-3) | 4.0 (3.9-4.5) | 0.70 |
| GFAP (pg/ml) | 323 | 3 (2-3) | 4.0 (3.9-4.5) | 209 | 3 (2-3) | 4.0 (3.8-4.5) | 114 | 3 (2-3) | 4.0 (3.9-4.5) | 0.70 |
| NfL (pg/ml) | 323 | 3 (2-3) | 4.0 (3.9-4.5) | 209 | 3 (2-3) | 4.0 (3.8-4.5) | 114 | 3 (2-3) | 4.0 (3.9-4.5) | 0.70 |

**Supplementary Table 3. Imaging and clinical follow-up time stratified by amyloid PET status.** The time from the first to the last data is presented as the median with the interquartile range in number of years. The significance of differences by amyloid PET status were evaluated with Wilcoxon’s rank-sum tests. All tests were two sided and not adjusted for covariates or multiple comparisons.

| **Characteristic** | **Full cohort** | | | **Amyloid PET negative** | | | **Amyloid PET positive** | | | **p=** |
| --- | --- | --- | --- | --- | --- | --- | --- | --- | --- | --- |
|  | **n=** | **o=** | **Follow-up**  **(years)** | **n=** | **o=** | **Follow-up**  **(years)** | **n=** | **o=** | **Follow-up**  **(years)** |  |
| Amyloid PET | 373 | 3 (3-3) | 4.1 (4.0-5.1) | 232 | 3 (3-3) | 4.2 (4.0-5.2) | 141 | 3 (3-3) | 4.1 (4.0-5.0) | 0.10 |
| Tau PET | 91 | 2 (2-3) | 2.0 (1.4-3.3) | 52 | 2 (2-3) | 2.0 (1.4-4.0) | 39 | 3 (2-3) | 2.0 (1.3-2.7) | 0.20 |
| Brain MRI | 314 | 4 (3-6) | 4.1 (2.3-5.1) | 202 | 4 (3-6) | 4.2 (2.8-5.2) | 112 | 4 (3-6) | 4.0 (2.1-5.1) | 0.08 |
| Clinical assessment | 373 | 5 (4-6) | 5.1 (4.2-5.5) | 232 | 5 (4-6) | 5.1 (4.3-5.5) | 141 | 6 (5-6) | 5.0 (4.1-5.3) | 0.20 |

**Supplementary Table 4. Correlations between baseline plasma biomarker levels and rates of change in amyloid PET Centiloid.** Unadjusted Spearman correlations are shown. Correlations between the top performing measure for each analyte and other measures were compared by bootstrapping.

**Full cohort**

| **Analyte** | **Measure** | **Spearman rho** | **p=** | **Comparison** |
| --- | --- | --- | --- | --- |
|  |  | **(95% CI)** |  |  |
| **Aβ42/Aβ40** | Roche NeuroToolKit plasma Aβ42/Aβ40 | -0.329 (-0.422 to -0.232) | <0.01 | REFERENCE |
|  | Fujirebio Lumipulse plasma Aβ42/Aβ40 | -0.310 (-0.403 to -0.209) | <0.01 | 0.69 |
|  | C2N Precivity plasma Aβ42/Aβ40 | -0.206 (-0.302 to -0.106) | <0.01 | 0.01 |
|  | Quanterix Neurology 4-Plex plasma Aβ42/Aβ40 | -0.204 (-0.305 to -0.102) | <0.01 | 0.05 |
| **p-tau217** | C2N Precivity plasma %p-tau217 | 0.299 (0.196 to 0.395) | <0.01 | REFERENCE |
|  | C2N Precivity plasma p-tau217 (pg/mL) | 0.285 (0.182 to 0.385) | <0.01 | 0.54 |
|  | Fujirebio Lumipulse plasma p-tau217 (pg/mL) | 0.236 (0.135 to 0.336) | <0.01 | 0.09 |
|  | Janssen LucentAD Quanterix plasma p-tau217 (pg/mL) | 0.209 (0.104 to 0.309) | <0.01 | 0.02 |
|  | ALZpath Quanterix plasma p-tau217 (pg/mL) | 0.207 (0.101 to 0.312) | <0.01 | 0.01 |
| **GFAP** | Quanterix Neurology 4-Plex plasma GFAP (pg/mL) | 0.170 (0.056 to 0.279) | <0.01 | REFERENCE |
|  | Roche NeuroToolKit plasma GFAP (ng/mL) | 0.169 (0.063 to 0.272) | <0.01 | 0.19 |
| **p-tau181** | Roche NeuroToolKit plasma p-tau181 (pg/mL) | 0.138 (0.031 to 0.238) | 0.01 | REFERENCE |
|  | Quanterix Neurology 4-Plex plasma p-tau181 (pg/ml) | 0.090 (-0.022 to 0.201) | 0.11 | 0.07 |
| **NfL** | Quanterix Neurology 4-Plex plasma NfL (pg/mL) | 0.091 (-0.021 to 0.201) | 0.10 | REFERENCE |
|  | Roche NeuroToolKit plasma NfL (pg/mL) | 0.078 (-0.028 to 0.185) | 0.13 | 0.55 |

**Amyloid PET positive sub-cohort**

| **Analyte** | **Measure** | **Spearman rho** | **p=** | **Comparison** |
| --- | --- | --- | --- | --- |
|  |  | **(95% CI)** |  |  |
| **p-tau217** | ALZpath Quanterix plasma p-tau217 (pg/mL) | -0.155 (-0.326 to 0.021) | 0.07 | REFERENCE |
|  | Janssen LucentAD Quanterix plasma p-tau217 (pg/mL) | -0.144 (-0.316 to 0.034) | 0.09 | 0.79 |
|  | Fujirebio Lumipulse plasma p-tau217 (pg/mL) | -0.135 (-0.299 to 0.038) | 0.11 | 0.64 |
|  | C2N Precivity plasma %p-tau217 | -0.129 (-0.305 to 0.048) | 0.13 | 0.66 |
|  | C2N Precivity plasma p-tau217 (pg/mL) | -0.063 (-0.234 to 0.112) | 0.46 | 0.13 |
| **Aβ42/Aβ40** | Fujirebio Lumipulse plasma Aβ42/Aβ40 | -0.114 (-0.283 to 0.059) | 0.18 | REFERENCE |
|  | Quanterix Neurology 4-Plex plasma Aβ42/Aβ40 | -0.110 (-0.271 to 0.064) | 0.24 | 0.56 |
|  | Roche NeuroToolKit plasma Aβ42/Aβ40 | -0.089 (-0.253 to 0.078) | 0.30 | 0.65 |
|  | C2N Precivity plasma Aβ42/Aβ40 | -0.051 (-0.211 to 0.105) | 0.55 | 0.45 |
| **p-tau181** | Roche NeuroToolKit plasma p-tau181 (pg/mL) | -0.093 (-0.268 to 0.082) | 0.28 | REFERENCE |
|  | Quanterix Neurology 4-Plex plasma p-tau181 (pg/ml) | -0.017 (-0.202 to 0.166) | 0.86 | 0.93 |
| **GFAP** | Quanterix Neurology 4-Plex plasma GFAP (pg/mL) | 0.044 (-0.153 to 0.235) | 0.64 | REFERENCE |
|  | Roche NeuroToolKit plasma GFAP (ng/mL) | 0.010 (-0.170 to 0.186) | 0.91 | 0.5 |
| **NfL** | Roche NeuroToolKit plasma NfL (pg/mL) | -0.039 (-0.209 to 0.132) | 0.64 | REFERENCE |
|  | Quanterix Neurology 4-Plex plasma NfL (pg/mL) | 0.021 (-0.170 to 0.214) | 0.82 | 0.84 |

**Amyloid PET negative sub-cohort**

| **Analyte** | **Measure** | **Spearman rho** | **p=** | **Comparison** |
| --- | --- | --- | --- | --- |
|  |  | **(95% CI)** |  |  |
| **Aβ42/Aβ40** | Roche NeuroToolKit plasma Aβ42/Aβ40 | -0.359 (-0.469 to -0.240) | <0.01 | REFERENCE |
|  | Fujirebio Lumipulse plasma Aβ42/Aβ40 | -0.293 (-0.409 to -0.169) | <0.01 | 0.26 |
|  | C2N Precivity plasma Aβ42/Aβ40 | -0.157 (-0.277 to -0.034) | 0.02 | 0.004 |
|  | Quanterix Neurology 4-Plex plasma Aβ42/Aβ40 | -0.150 (-0.277 to -0.019) | 0.03 | 0.0064 |
| **p-tau217** | C2N Precivity plasma %p-tau217 | 0.260 (0.128 to 0.382) | <0.01 | REFERENCE |
|  | C2N Precivity plasma p-tau217 (pg/mL) | 0.207 (0.075 to 0.335) | <0.01 | 0.26 |
|  | Fujirebio Lumipulse plasma p-tau217 (pg/mL) | 0.171 (0.042 to 0.297) | <0.01 | 0.29 |
|  | Janssen LucentAD Quanterix plasma p-tau217 (pg/mL) | 0.158 (0.026 to 0.286) | 0.02 | 0.23 |
|  | ALZpath Quanterix plasma p-tau217 (pg/mL) | 0.126 (-0.010 to 0.255) | 0.06 | 0.085 |
| **GFAP** | Roche NeuroToolKit plasma GFAP (ng/mL) | 0.120 (-0.013 to 0.254) | 0.07 | REFERENCE |
|  | Quanterix Neurology 4-Plex plasma GFAP (pg/mL) | 0.118 (-0.023 to 0.254) | 0.09 | 0.42 |
| **NfL** | Roche NeuroToolKit plasma NfL (pg/mL) | 0.067 (-0.071 to 0.201) | 0.31 | REFERENCE |
|  | Quanterix Neurology 4-Plex plasma NfL (pg/mL) | 0.043 (-0.097 to 0.182) | 0.54 | 0.29 |
| **p-tau181** | Quanterix Neurology 4-Plex plasma p-tau181 (pg/ml) | -0.021 (-0.165 to 0.116) | 0.76 | REFERENCE |
|  | Roche NeuroToolKit plasma p-tau181 (pg/mL) | 0.007 (-0.127 to 0.139) | 0.92 | 0.55 |

**Supplementary Table 5. Correlations between rates of change plasma biomarker levels and baseline biomarker levels with white matter adjusted amyloid PET SUVR.**Unadjusted Spearman correlations are shown. Correlations between the top performing measure for each analyte and other measures were compared by bootstrapping.

**Full cohort**

| **Analyte** | **Measure** | **Spearman rho** | **p=** | **Comparison** |
| --- | --- | --- | --- | --- |
|  |  | **(95% CI)** |  |  |
| **Aβ42/Aβ40** | Fujirebio Lumipulse plasma Aβ42/Aβ40 | -0.368 (-0.454 to -0.274) | <0.01 | REFERENCE |
|  | Roche NeuroToolKit plasma Aβ42/Aβ40 | -0.293 (-0.386 to -0.192) | <0.01 | 0.12 |
|  | Quanterix Neurology 4-Plex plasma Aβ42/Aβ40 | -0.282 (-0.387 to -0.173) | <0.01 | 0.04 |
|  | C2N Precivity plasma Aβ42/Aβ40 | -0.261 (-0.363 to -0.156) | <0.01 | 0.02 |
| **p-tau217** | C2N Precivity plasma %p-tau217 | 0.338 (0.248 to 0.421) | <0.01 | REFERENCE |
|  | C2N Precivity plasma p-tau217 (pg/mL) | 0.320 (0.228 to 0.411) | <0.01 | 0.28 |
|  | ALZpath Quanterix plasma p-tau217 (pg/mL) | 0.291 (0.193 to 0.386) | <0.01 | 0.2 |
|  | Janssen LucentAD Quanterix plasma p-tau217 (pg/mL) | 0.276 (0.180 to 0.368) | <0.01 | 0.01 |
|  | Fujirebio Lumipulse plasma p-tau217 (pg/mL) | 0.266 (0.166 to 0.360) | <0.01 | 0.05 |
| **p-tau181** | Roche NeuroToolKit plasma p-tau181 (pg/mL) | 0.208 (0.108 to 0.306) | <0.01 | REFERENCE |
|  | Quanterix Neurology 4-Plex plasma p-tau181 (pg/ml) | 0.152 (0.039 to 0.264) | <0.01 | 0.13 |
| **GFAP** | Quanterix Neurology 4-Plex plasma GFAP (pg/mL) | 0.157 (0.045 to 0.267) | <0.01 | REFERENCE |
|  | Roche NeuroToolKit plasma GFAP (ng/mL) | 0.127 (0.019 to 0.234) | 0.02 | 0.47 |
| **NfL** | Roche NeuroToolKit plasma NfL (pg/mL) | 0.099 (-0.011 to 0.207) | 0.07 | REFERENCE |
|  | Quanterix Neurology 4-Plex plasma NfL (pg/mL) | 0.094 (-0.023 to 0.209) | 0.11 | 0.95 |

**Amyloid PET positive sub-cohort**

| **Analyte** | **Measure** | **Spearman rho** | **p=** | **Comparison** |
| --- | --- | --- | --- | --- |
|  |  | **(95% CI)** |  |  |
| **p-tau217** | C2N Precivity plasma %p-tau217 | -0.202 (-0.363 to -0.033) | 0.02 | REFERENCE |
|  | Fujirebio Lumipulse plasma p-tau217 (pg/mL) | -0.173 (-0.341 to -0.001) | 0.05 | 0.62 |
|  | ALZpath Quanterix plasma p-tau217 (pg/mL) | -0.160 (-0.333 to 0.017) | 0.07 | 0.49 |
|  | Janssen LucentAD Quanterix plasma p-tau217 (pg/mL) | -0.144 (-0.310 to 0.028) | 0.11 | 0.34 |
|  | C2N Precivity plasma p-tau217 (pg/mL) | -0.138 (-0.299 to 0.030) | 0.12 | 0.10 |
| **p-tau181** | Quanterix Neurology 4-Plex plasma p-tau181 (pg/ml) | -0.130 (-0.328 to 0.069) | 0.20 | REFERENCE |
|  | Roche NeuroToolKit plasma p-tau181 (pg/mL) | -0.106 (-0.270 to 0.065) | 0.24 | 0.36 |
| **Aβ42/Aβ40** | C2N Precivity plasma Aβ42/Aβ40 | -0.128 (-0.303 to 0.049) | 0.15 | REFERENCE |
|  | Quanterix Neurology 4-Plex plasma Aβ42/Aβ40 | -0.112 (-0.302 to 0.092) | 0.27 | 0.63 |
|  | Fujirebio Lumipulse plasma Aβ42/Aβ40 | -0.058 (-0.228 to 0.115) | 0.52 | 0.40 |
|  | Roche NeuroToolKit plasma Aβ42/Aβ40 | 0.005 (-0.180 to 0.193) | 0.95 | 0.22 |
| **NfL** | Quanterix Neurology 4-Plex plasma NfL (pg/mL) | 0.124 (-0.086 to 0.325) | 0.22 | REFERENCE |
|  | Roche NeuroToolKit plasma NfL (pg/mL) | 0.113 (-0.067 to 0.286) | 0.21 | 0.53 |
| **GFAP** | Quanterix Neurology 4-Plex plasma GFAP (pg/mL) | 0.028 (-0.180 to 0.232) | 0.78 | REFERENCE |
|  | Roche NeuroToolKit plasma GFAP (ng/mL) | 0.002 (-0.185 to 0.183) | 0.98 | 0.33 |

**Amyloid PET negative sub-cohort**

| **Analyte** | **Measure** | **Spearman rho** | **p=** | **Comparison** |
| --- | --- | --- | --- | --- |
|  |  | **(95% CI)** |  |  |
| **Aβ42/Aβ40** | Fujirebio Lumipulse plasma Aβ42/Aβ40 | -0.279 (-0.400 to -0.150) | <0.01 | REFERENCE |
|  | Roche NeuroToolKit plasma Aβ42/Aβ40 | -0.210 (-0.337 to -0.073) | <0.01 | 0.31 |
|  | C2N Precivity plasma Aβ42/Aβ40 | -0.163 (-0.296 to -0.024) | 0.02 | 0.11 |
|  | Quanterix Neurology 4-Plex plasma Aβ42/Aβ40 | -0.145 (-0.283 to -0.001) | 0.05 | 0.12 |
| **p-tau217** | C2N Precivity plasma %p-tau217 | 0.225 (0.098 to 0.345) | <0.01 | REFERENCE |
|  | C2N Precivity plasma p-tau217 (pg/mL) | 0.195 (0.063 to 0.323) | <0.01 | 0.43 |
|  | Janssen LucentAD Quanterix plasma p-tau217 (pg/mL) | 0.195 (0.068 to 0.313) | <0.01 | 0.62 |
|  | ALZpath Quanterix plasma p-tau217 (pg/mL) | 0.194 (0.064 to 0.319) | <0.01 | 0.67 |
|  | Fujirebio Lumipulse plasma p-tau217 (pg/mL) | 0.138 (-0.001 to 0.270) | 0.04 | 0.23 |
| **p-tau181** | Roche NeuroToolKit plasma p-tau181 (pg/mL) | 0.090 (-0.042 to 0.218) | 0.19 | REFERENCE |
|  | Quanterix Neurology 4-Plex plasma p-tau181 (pg/ml) | 0.064 (-0.085 to 0.211) | 0.38 | 0.91 |
| **GFAP** | Roche NeuroToolKit plasma GFAP (ng/mL) | -0.067 (-0.206 to 0.074) | 0.33 | REFERENCE |
|  | Quanterix Neurology 4-Plex plasma GFAP (pg/mL) | -0.043 (-0.191 to 0.107) | 0.55 | 0.27 |
| **NfL** | Quanterix Neurology 4-Plex plasma NfL (pg/mL) | -0.045 (-0.186 to 0.098) | 0.54 | REFERENCE |
|  | Roche NeuroToolKit plasma NfL (pg/mL) | -0.033 (-0.165 to 0.101) | 0.63 | 0.48 |

**Supplementary Table 6. Correlations between rates of change plasma biomarker levels and baseline biomarker levels with composite reference adjusted amyloid PET SUVR.** Unadjusted Spearman correlations are shown. Correlations between the top performing measure for each analyte and other measures were compared by bootstrapping.

**Full cohort**

| **Analyte** | **Measure** | **Spearman rho** | **p=** | **Comparison** |
| --- | --- | --- | --- | --- |
|  |  | **(95% CI)** |  |  |
| **Aβ42/Aβ40** | Fujirebio Lumipulse plasma Aβ42/Aβ40 | -0.476 (-0.552 to -0.393) | <0.01 | REFERENCE |
|  | Roche NeuroToolKit plasma Aβ42/Aβ40 | -0.422 (-0.507 to -0.333) | <0.01 | 0.22 |
|  | Quanterix Neurology 4-Plex plasma Aβ42/Aβ40 | -0.354 (-0.450 to -0.251) | <0.01 | <0.01 |
|  | C2N Precivity plasma Aβ42/Aβ40 | -0.345 (-0.437 to -0.248) | <0.01 | <0.01 |
| **p-tau217** | C2N Precivity plasma %p-tau217 | 0.441 (0.353 to 0.522) | <0.01 | REFERENCE |
|  | C2N Precivity plasma p-tau217 (pg/mL) | 0.435 (0.342 to 0.520) | <0.01 | 0.64 |
|  | ALZpath Quanterix plasma p-tau217 (pg/mL) | 0.380 (0.287 to 0.467) | <0.01 | 0.07 |
|  | Fujirebio Lumipulse plasma p-tau217 (pg/mL) | 0.365 (0.270 to 0.455) | <0.01 | 0.03 |
|  | Janssen LucentAD Quanterix plasma p-tau217 (pg/mL) | 0.364 (0.271 to 0.452) | <0.01 | 0.03 |
| **p-tau181** | Roche NeuroToolKit plasma p-tau181 (pg/mL) | 0.281 (0.183 to 0.374) | <0.01 | REFERENCE |
|  | Quanterix Neurology 4-Plex plasma p-tau181 (pg/ml) | 0.220 (0.111 to 0.324) | <0.01 | 0.06 |
| **GFAP** | Quanterix Neurology 4-Plex plasma GFAP (pg/mL) | 0.236 (0.129 to 0.339) | <0.01 | REFERENCE |
|  | Roche NeuroToolKit plasma GFAP (ng/mL) | 0.223 (0.123 to 0.322) | <0.01 | 0.90 |
| **NfL** | Quanterix Neurology 4-Plex plasma NfL (pg/mL) | 0.135 (0.025 to 0.244) | 0.02 | REFERENCE |
|  | Roche NeuroToolKit plasma NfL (pg/mL) | 0.124 (0.023 to 0.225) | 0.02 | 0.98 |

**Amyloid PET positive sub-cohort**

| **Analyte** | **Measure** | **Spearman rho** | **p=** | **Comparison** |
| --- | --- | --- | --- | --- |
|  |  | **(95% CI)** |  |  |
| **Aβ42/Aβ40** | Quanterix Neurology 4-Plex plasma Aβ42/Aβ40 | -0.148 (-0.321 to 0.032) | 0.12 | REFERENCE |
|  | C2N Precivity plasma Aβ42/Aβ40 | -0.146 (-0.313 to 0.027) | 0.08 | 0.99 |
|  | Fujirebio Lumipulse plasma Aβ42/Aβ40 | -0.138 (-0.298 to 0.026) | 0.10 | 0.71 |
|  | Roche NeuroToolKit plasma Aβ42/Aβ40 | -0.100 (-0.265 to 0.065) | 0.24 | 0.65 |
| **p-tau217** | Fujirebio Lumipulse plasma p-tau217 (pg/mL) | -0.146 (-0.311 to 0.024) | 0.08 | REFERENCE |
|  | C2N Precivity plasma %p-tau217 | -0.139 (-0.310 to 0.041) | 0.10 | 0.89 |
|  | ALZpath Quanterix plasma p-tau217 (pg/mL) | -0.132 (-0.301 to 0.039) | 0.12 | 0.75 |
|  | Janssen LucentAD Quanterix plasma p-tau217 (pg/mL) | -0.113 (-0.286 to 0.061) | 0.18 | 0.47 |
|  | C2N Precivity plasma p-tau217 (pg/mL) | -0.050 (-0.220 to 0.122) | 0.55 | 0.13 |
| **GFAP** | Quanterix Neurology 4-Plex plasma GFAP (pg/mL) | 0.077 (-0.111 to 0.259) | 0.42 | REFERENCE |
|  | Roche NeuroToolKit plasma GFAP (ng/mL) | 0.056 (-0.118 to 0.223) | 0.51 | 0.19 |
| **p-tau181** | Roche NeuroToolKit plasma p-tau181 (pg/mL) | -0.070 (-0.233 to 0.098) | 0.41 | REFERENCE |
|  | Quanterix Neurology 4-Plex plasma p-tau181 (pg/ml) | -0.047 (-0.243 to 0.144) | 0.62 | 0.78 |
| **NfL** | Quanterix Neurology 4-Plex plasma NfL (pg/mL) | 0.056 (-0.133 to 0.248) | 0.55 | REFERENCE |
|  | Roche NeuroToolKit plasma NfL (pg/mL) | 0.027 (-0.139 to 0.190) | 0.75 | 0.46 |

**Amyloid PET negative sub-cohort**

| **Analyte** | **Measure** | **Spearman rho** | **p=** | **Comparison** |
| --- | --- | --- | --- | --- |
|  |  | **(95% CI)** |  |  |
| **Aβ42/Aβ40** | Fujirebio Lumipulse plasma Aβ42/Aβ40 | -0.390 (-0.497 to -0.272) | <0.01 | REFERENCE |
|  | Roche NeuroToolKit plasma Aβ42/Aβ40 | -0.348 (-0.468 to -0.219) | <0.01 | 0.54 |
|  | Quanterix Neurology 4-Plex plasma Aβ42/Aβ40 | -0.264 (-0.385 to -0.133) | <0.01 | 0.06 |
|  | C2N Precivity plasma Aβ42/Aβ40 | -0.255 (-0.379 to -0.129) | <0.01 | 0.06 |
| **p-tau217** | C2N Precivity plasma %p-tau217 | 0.332 (0.211 to 0.443) | <0.01 | REFERENCE |
|  | C2N Precivity plasma p-tau217 (pg/mL) | 0.317 (0.198 to 0.430) | <0.01 | 0.68 |
|  | Janssen LucentAD Quanterix plasma p-tau217 (pg/mL) | 0.276 (0.158 to 0.389) | <0.01 | 0.42 |
|  | ALZpath Quanterix plasma p-tau217 (pg/mL) | 0.274 (0.149 to 0.395) | <0.01 | 0.45 |
|  | Fujirebio Lumipulse plasma p-tau217 (pg/mL) | 0.229 (0.099 to 0.350) | <0.01 | 0.17 |
| **p-tau181** | Roche NeuroToolKit plasma p-tau181 (pg/mL) | 0.126 (-0.003 to 0.252) | 0.06 | REFERENCE |
|  | Quanterix Neurology 4-Plex plasma p-tau181 (pg/ml) | 0.094 (-0.046 to 0.235) | 0.18 | 0.86 |
| **GFAP** | Quanterix Neurology 4-Plex plasma GFAP (pg/mL) | 0.066 (-0.075 to 0.203) | 0.34 | REFERENCE |
|  | Roche NeuroToolKit plasma GFAP (ng/mL) | 0.043 (-0.094 to 0.179) | 0.51 | 0.44 |
| **NfL** | Quanterix Neurology 4-Plex plasma NfL (pg/mL) | 0.031 (-0.109 to 0.171) | 0.66 | REFERENCE |
|  | Roche NeuroToolKit plasma NfL (pg/mL) | 0.022 (-0.108 to 0.152) | 0.73 | 0.46 |

**Supplementary Table 7. Correlations between baseline plasma biomarker levels and rates of change in early tau PET.** Unadjusted Spearman correlations are shown. Correlations between the top performing measure for each analyte and other measures were compared by bootstrapping.

**Full cohort**

| **Analyte** | **Measure** | **Spearman rho** | **p=** | **Comparison** |
| --- | --- | --- | --- | --- |
|  |  | **(95% CI)** |  |  |
| **Aβ42/Aβ40** | Fujirebio Lumipulse plasma Aβ42/Aβ40 | -0.348 (-0.513 to -0.155) | <0.01 | REFERENCE |
|  | Roche NeuroToolKit plasma Aβ42/Aβ40 | -0.174 (-0.360 to 0.027) | 0.10 | 0.061 |
|  | C2N Precivity plasma Aβ42/Aβ40 | -0.162 (-0.350 to 0.036) | 0.12 | 0.026 |
|  | Quanterix Neurology 4-Plex plasma Aβ42/Aβ40 | -0.125 (-0.346 to 0.105) | 0.31 | 0.042 |
| **p-tau217** | C2N Precivity plasma p-tau217 (pg/mL) | 0.237 (0.011 to 0.450) | 0.02 | REFERENCE |
|  | C2N Precivity plasma %p-tau217 | 0.237 (0.017 to 0.443) | 0.02 | 1 |
|  | Fujirebio Lumipulse plasma p-tau217 (pg/mL) | 0.154 (-0.055 to 0.359) | 0.14 | 0.23 |
|  | ALZpath Quanterix plasma p-tau217 (pg/mL) | 0.113 (-0.108 to 0.337) | 0.29 | 0.093 |
|  | Janssen LucentAD Quanterix plasma p-tau217 (pg/mL) | 0.087 (-0.140 to 0.311) | 0.41 | 0.086 |
| **NfL** | Roche NeuroToolKit plasma NfL (pg/mL) | -0.216 (-0.416 to 0.003) | 0.04 | REFERENCE |
|  | Quanterix Neurology 4-Plex plasma NfL (pg/mL) | -0.184 (-0.433 to 0.086) | 0.13 | 0.24 |
| **GFAP** | Roche NeuroToolKit plasma GFAP (ng/mL) | -0.117 (-0.319 to 0.085) | 0.27 | REFERENCE |
|  | Quanterix Neurology 4-Plex plasma GFAP (pg/mL) | -0.027 (-0.271 to 0.218) | 0.82 | 0.22 |
| **p-tau181** | Quanterix Neurology 4-Plex plasma p-tau181 (pg/ml) | 0.114 (-0.155 to 0.383) | 0.35 | REFERENCE |
|  | Roche NeuroToolKit plasma p-tau181 (pg/mL) | 0.074 (-0.156 to 0.301) | 0.48 | 0.99 |

**Amyloid PET positive sub-cohort**

| **Analyte** | **Measure** | **Spearman rho** | **p=** | **Comparison** |
| --- | --- | --- | --- | --- |
|  |  | **(95% CI)** |  |  |
| **Aβ42/Aβ40** | Fujirebio Lumipulse plasma Aβ42/Aβ40 | -0.354 (-0.611 to -0.029) | 0.03 | REFERENCE |
|  | Roche NeuroToolKit plasma Aβ42/Aβ40 | -0.215 (-0.504 to 0.120) | 0.19 | 0.42 |
|  | C2N Precivity plasma Aβ42/Aβ40 | -0.122 (-0.415 to 0.192) | 0.46 | 0.16 |
|  | Quanterix Neurology 4-Plex plasma Aβ42/Aβ40 | -0.081 (-0.458 to 0.303) | 0.70 | 0.11 |
| **NfL** | Roche NeuroToolKit plasma NfL (pg/mL) | -0.206 (-0.494 to 0.129) | 0.21 | REFERENCE |
|  | Quanterix Neurology 4-Plex plasma NfL (pg/mL) | -0.145 (-0.580 to 0.359) | 0.49 | 0.56 |
| **GFAP** | Quanterix Neurology 4-Plex plasma GFAP (pg/mL) | 0.168 (-0.272 to 0.601) | 0.42 | REFERENCE |
|  | Roche NeuroToolKit plasma GFAP (ng/mL) | 0.102 (-0.236 to 0.418) | 0.54 | 0.64 |
| **p-tau181** | Quanterix Neurology 4-Plex plasma p-tau181 (pg/ml) | 0.125 (-0.342 to 0.564) | 0.55 | REFERENCE |
|  | Roche NeuroToolKit plasma p-tau181 (pg/mL) | -0.074 (-0.434 to 0.306) | 0.66 | 0.69 |
| **p-tau217** | C2N Precivity plasma %p-tau217 | 0.098 (-0.258 to 0.439) | 0.55 | REFERENCE |
|  | Janssen LucentAD Quanterix plasma p-tau217 (pg/mL) | 0.093 (-0.271 to 0.455) | 0.57 | 0.95 |
|  | C2N Precivity plasma p-tau217 (pg/mL) | 0.092 (-0.268 to 0.444) | 0.58 | 0.92 |
|  | ALZpath Quanterix plasma p-tau217 (pg/mL) | -0.031 (-0.392 to 0.345) | 0.85 | 0.63 |
|  | Fujirebio Lumipulse plasma p-tau217 (pg/mL) | -0.007 (-0.354 to 0.356) | 0.96 | 0.44 |

**Amyloid PET negative sub-cohort**

| **Analyte** | **Measure** | **Spearman rho** | **p=** | **Comparison** |
| --- | --- | --- | --- | --- |
|  |  | **(95% CI)** |  |  |
| **Aβ42/Aβ40** | Fujirebio Lumipulse plasma Aβ42/Aβ40 | -0.364 (-0.588 to -0.089) | 0.01 | REFERENCE |
|  | C2N Precivity plasma Aβ42/Aβ40 | -0.214 (-0.451 to 0.048) | 0.13 | 0.19 |
|  | Roche NeuroToolKit plasma Aβ42/Aβ40 | -0.169 (-0.411 to 0.093) | 0.23 | 0.098 |
|  | Quanterix Neurology 4-Plex plasma Aβ42/Aβ40 | -0.166 (-0.455 to 0.144) | 0.28 | 0.19 |
| **p-tau217** | C2N Precivity plasma %p-tau217 | 0.339 (0.054 to 0.586) | 0.01 | REFERENCE |
|  | C2N Precivity plasma p-tau217 (pg/mL) | 0.327 (0.050 to 0.573) | 0.02 | 0.88 |
|  | Fujirebio Lumipulse plasma p-tau217 (pg/mL) | 0.121 (-0.158 to 0.383) | 0.39 | 0.15 |
|  | ALZpath Quanterix plasma p-tau217 (pg/mL) | 0.118 (-0.166 to 0.405) | 0.41 | 0.11 |
|  | Janssen LucentAD Quanterix plasma p-tau217 (pg/mL) | -0.106 (-0.370 to 0.165) | 0.45 | 0.21 |
| **GFAP** | Roche NeuroToolKit plasma GFAP (ng/mL) | -0.329 (-0.533 to -0.087) | 0.02 | REFERENCE |
|  | Quanterix Neurology 4-Plex plasma GFAP (pg/mL) | -0.217 (-0.486 to 0.093) | 0.16 | 0.12 |
| **NfL** | Roche NeuroToolKit plasma NfL (pg/mL) | -0.269 (-0.533 to 0.033) | 0.05 | REFERENCE |
|  | Quanterix Neurology 4-Plex plasma NfL (pg/mL) | -0.250 (-0.540 to 0.079) | 0.1 | 0.22 |
| **p-tau181** | Quanterix Neurology 4-Plex plasma p-tau181 (pg/ml) | 0.074 (-0.268 to 0.420) | 0.63 | REFERENCE |
|  | Roche NeuroToolKit plasma p-tau181 (pg/mL) | 0.026 (-0.259 to 0.322) | 0.86 | 0.59 |

**Supplementary Table 8. Correlations between baseline plasma biomarker levels and rates of change in late tau PET.** Unadjusted Spearman correlations are shown. Correlations between the top performing measure for each analyte and other measures were compared by bootstrapping.

**Full cohort**

| **Analyte** | **Measure** | **Spearman rho** | **p=** | **Comparison** |
| --- | --- | --- | --- | --- |
|  |  | **(95% CI)** |  |  |
| **Aβ42/Aβ40** | Fujirebio Lumipulse plasma Aβ42/Aβ40 | -0.240 (-0.419 to -0.049) | 0.02 | REFERENCE |
|  | Roche NeuroToolKit plasma Aβ42/Aβ40 | -0.231 (-0.413 to -0.034) | 0.03 | 0.93 |
|  | C2N Precivity plasma Aβ42/Aβ40 | -0.116 (-0.302 to 0.080) | 0.27 | 0.16 |
|  | Quanterix Neurology 4-Plex plasma Aβ42/Aβ40 | -0.086 (-0.319 to 0.151) | 0.48 | 0.072 |
| **p-tau217** | C2N Precivity plasma p-tau217 (pg/mL) | 0.232 (0.016 to 0.431) | 0.03 | REFERENCE |
|  | C2N Precivity plasma %p-tau217 | 0.217 (0.005 to 0.413) | 0.04 | 0.7 |
|  | ALZpath Quanterix plasma p-tau217 (pg/mL) | 0.201 (-0.023 to 0.404) | 0.06 | 0.63 |
|  | Fujirebio Lumipulse plasma p-tau217 (pg/mL) | 0.198 (-0.016 to 0.408) | 0.06 | 0.63 |
|  | Janssen LucentAD Quanterix plasma p-tau217 (pg/mL) | 0.158 (-0.054 to 0.361) | 0.13 | 0.28 |
| **p-tau181** | Quanterix Neurology 4-Plex plasma p-tau181 (pg/ml) | 0.212 (-0.038 to 0.445) | 0.08 | REFERENCE |
|  | Roche NeuroToolKit plasma p-tau181 (pg/mL) | 0.186 (-0.042 to 0.403) | 0.08 | 0.99 |
| **GFAP** | Quanterix Neurology 4-Plex plasma GFAP (pg/mL) | 0.067 (-0.174 to 0.308) | 0.59 | REFERENCE |
|  | Roche NeuroToolKit plasma GFAP (ng/mL) | -0.024 (-0.234 to 0.190) | 0.82 | 0.65 |
| **NfL** | Roche NeuroToolKit plasma NfL (pg/mL) | -0.023 (-0.237 to 0.195) | 0.83 | REFERENCE |
|  | Quanterix Neurology 4-Plex plasma NfL (pg/mL) | -0.017 (-0.266 to 0.241) | 0.89 | 0.7 |

**Amyloid PET positive sub-cohort**

| **Analyte** | **Measure** | **Spearman rho** | **p=** | **Comparison** |
| --- | --- | --- | --- | --- |
|  |  | **(95% CI)** |  |  |
| **Aβ42/Aβ40** | Roche NeuroToolKit plasma Aβ42/Aβ40 | -0.382 (-0.645 to -0.061) | 0.02 | REFERENCE |
|  | Fujirebio Lumipulse plasma Aβ42/Aβ40 | -0.189 (-0.490 to 0.149) | 0.25 | 0.26 |
|  | Quanterix Neurology 4-Plex plasma Aβ42/Aβ40 | -0.042 (-0.482 to 0.401) | 0.84 | 0.063 |
|  | C2N Precivity plasma Aβ42/Aβ40 | -0.033 (-0.339 to 0.280) | 0.84 | 0.036 |
| **p-tau181** | Quanterix Neurology 4-Plex plasma p-tau181 (pg/ml) | 0.342 (-0.083 to 0.675) | 0.09 | REFERENCE |
|  | Roche NeuroToolKit plasma p-tau181 (pg/mL) | 0.200 (-0.159 to 0.520) | 0.22 | 0.99 |
| **p-tau217** | C2N Precivity plasma p-tau217 (pg/mL) | 0.194 (-0.163 to 0.524) | 0.24 | REFERENCE |
|  | ALZpath Quanterix plasma p-tau217 (pg/mL) | 0.187 (-0.151 to 0.509) | 0.25 | 0.93 |
|  | Janssen LucentAD Quanterix plasma p-tau217 (pg/mL) | 0.178 (-0.171 to 0.499) | 0.28 | 0.84 |
|  | Fujirebio Lumipulse plasma p-tau217 (pg/mL) | 0.131 (-0.222 to 0.488) | 0.43 | 0.59 |
|  | C2N Precivity plasma %p-tau217 | 0.103 (-0.241 to 0.438) | 0.53 | 0.32 |
| **GFAP** | Quanterix Neurology 4-Plex plasma GFAP (pg/mL) | 0.152 (-0.286 to 0.533) | 0.47 | REFERENCE |
|  | Roche NeuroToolKit plasma GFAP (ng/mL) | 0.010 (-0.339 to 0.352) | 0.95 | 0.48 |
| **NfL** | Roche NeuroToolKit plasma NfL (pg/mL) | 0.015 (-0.333 to 0.358) | 0.93 | REFERENCE |
|  | Quanterix Neurology 4-Plex plasma NfL (pg/mL) | -0.002 (-0.478 to 0.466) | 1 | 0.34 |

**Amyloid PET negative sub-cohort**

| **Analyte** | **Measure** | **Spearman rho** | **p=** | **Comparison** |
| --- | --- | --- | --- | --- |
|  |  | **(95% CI)** |  |  |
| **GFAP** | Roche NeuroToolKit plasma GFAP (ng/mL) | -0.199 (-0.444 to 0.064) | 0.16 | REFERENCE |
|  | Quanterix Neurology 4-Plex plasma GFAP (pg/mL) | -0.097 (-0.389 to 0.214) | 0.53 | 0.21 |
| **p-tau217** | Janssen LucentAD Quanterix plasma p-tau217 (pg/mL) | -0.187 (-0.446 to 0.097) | 0.19 | REFERENCE |
|  | C2N Precivity AD2 plasma p-tau217 (pg/mL) | -0.031 (-0.313 to 0.257) | 0.83 | 0.25 |
|  | C2N Precivity AD2 plasma %p-tau217 | 0.013 (-0.282 to 0.295) | 0.93 | 0.24 |
|  | Fujirebio Lumipulse plasma p-tau217 (pg/mL) | -0.009 (-0.306 to 0.285) | 0.95 | 0.21 |
|  | ALZpath Quanterix plasma p-tau217 (pg/mL) | -0.001 (-0.280 to 0.286) | 0.99 | 0.18 |
| **NfL** | Roche NeuroToolKit plasma NfL (pg/mL) | -0.170 (-0.461 to 0.133) | 0.23 | REFERENCE |
|  | Quanterix Neurology 4-Plex plasma NfL (pg/mL) | -0.163 (-0.468 to 0.167) | 0.29 | 0.58 |
| **Aβ42/Aβ40** | Fujirebio Lumipulse plasma Aβ42/Aβ40 | -0.086 (-0.350 to 0.193) | 0.54 | REFERENCE |
|  | Roche NeuroToolKit plasma Aβ42/Aβ40 | 0.045 (-0.215 to 0.301) | 0.75 | 0.73 |
|  | C2N Precivity AD2 plasma Aβ42/Aβ40 | -0.032 (-0.286 to 0.230) | 0.82 | 0.58 |
|  | Quanterix Neurology 4-Plex plasma Aβ42/Aβ40 | 0.031 (-0.267 to 0.324) | 0.84 | 0.37 |
| **p-tau181** | Roche NeuroToolKit plasma p-tau181 (pg/mL) | -0.055 (-0.350 to 0.246) | 0.7 | REFERENCE |
|  | Quanterix Neurology 4-Plex plasma p-tau181 (pg/ml) | 0.036 (-0.279 to 0.353) | 0.82 | 0.95 |

**Supplementary Table 9. Correlations between baseline plasma biomarker levels and rates of change in cortical thickness.** Unadjusted Spearman correlations are shown. Correlations between the top performing measure for each analyte and other measures were compared by bootstrapping.

**Full cohort**

| **Analyte** | **Measure** | **Spearman rho** | **p=** | **Comparison** |
| --- | --- | --- | --- | --- |
|  |  | **(95% CI)** |  |  |
| **p-tau217** | C2N Precivity plasma p-tau217 (pg/mL) | -0.301 (-0.407 to -0.190) | <0.01 | REFERENCE |
|  | C2N Precivity plasma %p-tau217 | -0.286 (-0.398 to -0.170) | <0.01 | 0.62 |
|  | ALZpath Quanterix plasma p-tau217 (pg/mL) | -0.284 (-0.389 to -0.173) | <0.01 | 0.64 |
|  | Janssen LucentAD Quanterix plasma p-tau217 (pg/mL) | -0.280 (-0.388 to -0.163) | <0.01 | 0.55 |
|  | Fujirebio Lumipulse plasma p-tau217 (pg/mL) | -0.253 (-0.366 to -0.137) | <0.01 | 0.18 |
| **p-tau181** | Roche NeuroToolKit plasma p-tau181 (pg/mL) | -0.228 (-0.336 to -0.114) | <0.01 | REFERENCE |
|  | Quanterix Neurology 4-Plex plasma p-tau181 (pg/ml) | -0.217 (-0.336 to -0.094) | <0.01 | 0.22 |
| **NfL** | Roche NeuroToolKit plasma NfL (pg/mL) | -0.177 (-0.288 to -0.064) | <0.01 | REFERENCE |
|  | Quanterix Neurology 4-Plex plasma NfL (pg/mL) | -0.135 (-0.251 to -0.012) | 0.03 | 0.45 |
| **Aβ42/Aβ40** | Fujirebio Lumipulse plasma Aβ42/Aβ40 | 0.164 (0.050 to 0.273) | <0.01 | REFERENCE |
|  | Roche NeuroToolKit plasma Aβ42/Aβ40 | 0.146 (0.031 to 0.256) | 0.01 | 0.79 |
|  | Quanterix Neurology 4-Plex plasma Aβ42/Aβ40 | 0.102 (-0.018 to 0.220) | 0.09 | 0.43 |
|  | C2N Precivity plasma Aβ42/Aβ40 | 0.072 (-0.042 to 0.183) | 0.20 | 0.065 |
| **GFAP** | Roche NeuroToolKit plasma GFAP (ng/mL) | -0.154 (-0.263 to -0.042) | 0.01 | REFERENCE |
|  | Quanterix Neurology 4-Plex plasma GFAP (pg/mL) | -0.089 (-0.211 to 0.036) | 0.15 | 0.64 |

**Amyloid PET positive sub-cohort**

| **Analyte** | **Measure** | **Spearman rho** | **p=** | **Comparison** |
| --- | --- | --- | --- | --- |
|  |  | **(95% CI)** |  |  |
| **p-tau217** | Janssen LucentAD Quanterix plasma p-tau217 (pg/mL) | -0.384 (-0.543 to -0.206) | <0.01 | REFERENCE |
|  | ALZpath Quanterix plasma p-tau217 (pg/mL) | -0.340 (-0.506 to -0.156) | <0.01 | 0.24 |
|  | Fujirebio Lumipulse plasma p-tau217 (pg/mL) | -0.340 (-0.505 to -0.150) | <0.01 | 0.42 |
|  | C2N Precivity plasma %p-tau217 | -0.336 (-0.500 to -0.152) | <0.01 | 0.46 |
|  | C2N Precivity plasma p-tau217 (pg/mL) | -0.318 (-0.480 to -0.138) | <0.01 | 0.18 |
| **p-tau181** | Roche NeuroToolKit plasma p-tau181 (pg/mL) | -0.380 (-0.540 to -0.204) | <0.01 | REFERENCE |
|  | Quanterix Neurology 4-Plex plasma p-tau181 (pg/ml) | -0.344 (-0.537 to -0.128) | <0.01 | 0.58 |
| **NfL** | Roche NeuroToolKit plasma NfL (pg/mL) | -0.289 (-0.451 to -0.113) | <0.01 | REFERENCE |
|  | Quanterix Neurology 4-Plex plasma NfL (pg/mL) | -0.236 (-0.423 to -0.037) | 0.03 | 0.67 |
| **GFAP** | Roche NeuroToolKit plasma GFAP (ng/mL) | -0.217 (-0.390 to -0.030) | 0.02 | REFERENCE |
|  | Quanterix Neurology 4-Plex plasma GFAP (pg/mL) | -0.194 (-0.388 to 0.016) | 0.07 | 0.78 |
| **Aβ42/Aβ40** | Quanterix Neurology 4-Plex plasma Aβ42/Aβ40 | 0.118 (-0.093 to 0.317) | 0.27 | REFERENCE |
|  | Fujirebio Lumipulse plasma Aβ42/Aβ40 | 0.100 (-0.103 to 0.294) | 0.29 | 0.48 |
|  | C2N Precivity plasma Aβ42/Aβ40 | 0.035 (-0.157 to 0.222) | 0.72 | 0.88 |
|  | Roche NeuroToolKit plasma Aβ42/Aβ40 | -0.011 (-0.203 to 0.182) | 0.91 | 0.36 |

**Amyloid PET negative sub-cohort**

| **Analyte** | **Measure** | **Spearman rho** | **p=** | **Comparison** |
| --- | --- | --- | --- | --- |
|  |  | **(95% CI)** |  |  |
| **p-tau217** | C2N Precivity plasma p-tau217 (pg/mL) | -0.124 (-0.263 to 0.018) | 0.08 | REFERENCE |
|  | C2N Precivity plasma %p-tau217 | -0.112 (-0.253 to 0.031) | 0.11 | 0.88 |
|  | ALZpath Quanterix plasma p-tau217 (pg/mL) | -0.074 (-0.213 to 0.070) | 0.30 | 0.44 |
|  | Janssen LucentAD Quanterix plasma p-tau217 (pg/mL) | -0.070 (-0.214 to 0.075) | 0.32 | 0.43 |
|  | Fujirebio Lumipulse plasma p-tau217 (pg/mL) | -0.044 (-0.188 to 0.105) | 0.53 | 0.23 |
| **GFAP** | Quanterix Neurology 4-Plex plasma GFAP (pg/mL) | 0.082 (-0.070 to 0.229) | 0.27 | REFERENCE |
|  | Roche NeuroToolKit plasma GFAP (ng/mL) | 0.036 (-0.107 to 0.173) | 0.61 | 0.8 |
| **Aβ42/Aβ40** | Roche NeuroToolKit plasma Aβ42/Aβ40 | 0.070 (-0.070 to 0.211) | 0.32 | REFERENCE |
|  | C2N Precivity plasma Aβ42/Aβ40 | -0.061 (-0.199 to 0.080) | 0.39 | 0.93 |
|  | Quanterix Neurology 4-Plex plasma Aβ42/Aβ40 | -0.035 (-0.176 to 0.112) | 0.64 | 0.87 |
|  | Fujirebio Lumipulse plasma Aβ42/Aβ40 | -0.025 (-0.169 to 0.118) | 0.73 | 0.58 |
| **p-tau181** | Quanterix Neurology 4-Plex plasma p-tau181 (pg/ml) | -0.063 (-0.208 to 0.086) | 0.40 | REFERENCE |
|  | Roche NeuroToolKit plasma p-tau181 (pg/mL) | -0.005 (-0.145 to 0.137) | 0.95 | 0.89 |
| **NfL** | Roche NeuroToolKit plasma NfL (pg/mL) | -0.041 (-0.182 to 0.104) | 0.56 | REFERENCE |
|  | Quanterix Neurology 4-Plex plasma NfL (pg/mL) | -0.035 (-0.187 to 0.115) | 0.64 | 0.68 |

**Supplementary Table 10. Correlations between baseline plasma biomarker levels and rates of change in CDR-SB.** Unadjusted Spearman correlations are shown. Correlations between the top performing measure for each analyte and other measures were compared by bootstrapping.

**Full cohort**

| **Analyte** | **Measure** | **Spearman rho**  **(95% CI)** | **p=** | **Comparison** |
| --- | --- | --- | --- | --- |
| **p-tau217** | ALZpath Quanterix plasma p-tau217 (pg/mL) | 0.437 (0.342 to 0.524) | <0.01 | REFERENCE |
|  | C2N Precivity plasma p-tau217 (pg/mL) | 0.436 (0.341 to 0.524) | <0.01 | 0.97 |
|  | C2N Precivity plasma %p-tau217 | 0.407 (0.311 to 0.496) | <0.01 | 0.37 |
|  | Fujirebio Lumipulse plasma p-tau217 (pg/mL) | 0.402 (0.305 to 0.492) | <0.01 | 0.18 |
|  | Janssen LucentAD Quanterix plasma p-tau217 (pg/mL) | 0.397 (0.300 to 0.488) | <0.01 | 0.061 |
| **p-tau181** | Roche NeuroToolKit plasma p-tau181 (pg/mL) | 0.385 (0.289 to 0.476) | <0.01 | REFERENCE |
|  | Quanterix Neurology 4-Plex plasma p-tau181 (pg/ml) | 0.353 (0.249 to 0.448) | <0.01 | 0.49 |
| **Aβ42/Aβ40** | Fujirebio Lumipulse plasma Aβ42/Aβ40 | -0.345 (-0.433 to -0.252) | <0.01 | REFERENCE |
|  | C2N Precivity plasma Aβ42/Aβ40 | -0.274 (-0.365 to -0.178) | <0.01 | 0.12 |
|  | Quanterix Neurology 4-Plex plasma Aβ42/Aβ40 | -0.250 (-0.353 to -0.141) | <0.01 | 0.0041 |
|  | Roche NeuroToolKit plasma Aβ42/Aβ40 | -0.194 (-0.287 to -0.094) | <0.01 | 0.00032 |
| **GFAP** | Roche NeuroToolKit plasma GFAP (ng/mL) | 0.278 (0.179 to 0.374) | <0.01 | REFERENCE |
|  | Quanterix Neurology 4-Plex plasma GFAP (pg/mL) | 0.258 (0.151 to 0.358) | <0.01 | 0.56 |
| **NfL** | Roche NeuroToolKit plasma NfL (pg/mL) | 0.271 (0.171 to 0.366) | <0.01 | REFERENCE |
|  | Quanterix Neurology 4-Plex plasma NfL (pg/mL) | 0.263 (0.158 to 0.362) | <0.01 | 0.97 |

**Amyloid PET positive sub-cohort**

| **Analyte** | **Measure** | **Spearman rho** | **p=** | **Comparison** |
| --- | --- | --- | --- | --- |
|  |  | **(95% CI)** |  |  |
| **p-tau217** | C2N Precivity plasma %p-tau217 | 0.504 (0.360 to 0.627) | <0.01 | REFERENCE |
|  | Fujirebio Lumipulse plasma p-tau217 (pg/mL) | 0.493 (0.353 to 0.618) | <0.01 | 0.85 |
|  | C2N Precivity plasma p-tau217 (pg/mL) | 0.462 (0.313 to 0.587) | <0.01 | 0.24 |
|  | Janssen LucentAD Quanterix plasma p-tau217 (pg/mL) | 0.461 (0.309 to 0.592) | <0.01 | 0.45 |
|  | ALZpath Quanterix plasma p-tau217 (pg/mL) | 0.457 (0.314 to 0.580) | <0.01 | 0.37 |
| **p-tau181** | Roche NeuroToolKit plasma p-tau181 (pg/mL) | 0.459 (0.321 to 0.582) | <0.01 | REFERENCE |
|  | Quanterix Neurology 4-Plex plasma p-tau181 (pg/ml) | 0.332 (0.159 to 0.490) | <0.01 | 0.11 |
| **NfL** | Roche NeuroToolKit plasma NfL (pg/mL) | 0.254 (0.087 to 0.413) | <0.01 | REFERENCE |
|  | Quanterix Neurology 4-Plex plasma NfL (pg/mL) | 0.222 (0.035 to 0.398) | 0.02 | 0.83 |
| **Aβ42/Aβ40** | Fujirebio Lumipulse plasma Aβ42/Aβ40 | -0.243 (-0.395 to -0.082) | <0.01 | REFERENCE |
|  | Quanterix Neurology 4-Plex plasma Aβ42/Aβ40 | -0.188 (-0.369 to 0.001) | 0.05 | 0.079 |
|  | C2N Precivity plasma Aβ42/Aβ40 | -0.137 (-0.292 to 0.028) | 0.11 | 0.24 |
|  | Roche NeuroToolKit plasma Aβ42/Aβ40 | -0.012 (-0.179 to 0.156) | 0.89 | 0.019 |
| **GFAP** | Quanterix Neurology 4-Plex plasma GFAP (pg/mL) | 0.198 (0.022 to 0.365) | 0.04 | REFERENCE |
|  | Roche NeuroToolKit plasma GFAP (ng/mL) | 0.193 (0.035 to 0.345) | 0.02 | 0.99 |

**Amyloid PET negative sub-cohort**

| **Analyte** | **Measure** | **Spearman rho** | **p=** | **Comparison** |
| --- | --- | --- | --- | --- |
|  |  | **(95% CI)** |  |  |
| **p-tau181** | Quanterix Neurology 4-Plex plasma p-tau181 (pg/ml) | 0.216 (0.075 to 0.349) | <0.01 | REFERENCE |
|  | Roche NeuroToolKit plasma p-tau181 (pg/mL) | 0.126 (-0.009 to 0.257) | 0.06 | 0.18 |
| **p-tau217** | ALZpath Quanterix plasma p-tau217 (pg/mL) | 0.205 (0.074 to 0.334) | <0.01 | REFERENCE |
|  | C2N Precivity plasma p-tau217 (pg/mL) | 0.162 (0.029 to 0.289) | 0.01 | 0.5 |
|  | Fujirebio Lumipulse plasma p-tau217 (pg/mL) | 0.116 (-0.015 to 0.243) | 0.08 | 0.12 |
|  | Janssen LucentAD Quanterix plasma p-tau217 (pg/mL) | 0.113 (-0.023 to 0.250) | 0.09 | 0.03 |
|  | C2N Precivity plasma %p-tau217 | 0.081 (-0.050 to 0.206) | 0.22 | 0.095 |
| **NfL** | Quanterix Neurology 4-Plex plasma NfL (pg/mL) | 0.163 (0.026 to 0.293) | 0.02 | REFERENCE |
|  | Roche NeuroToolKit plasma NfL (pg/mL) | 0.151 (0.018 to 0.281) | 0.02 | 0.71 |
| **Aβ42/Aβ40** | Fujirebio Lumipulse plasma Aβ42/Aβ40 | -0.143 (-0.266 to -0.014) | 0.03 | REFERENCE |
|  | C2N Precivity plasma Aβ42/Aβ40 | -0.115 (-0.240 to 0.014) | 0.08 | 0.66 |
|  | Quanterix Neurology 4-Plex plasma Aβ42/Aβ40 | -0.096 (-0.230 to 0.038) | 0.17 | 0.33 |
|  | Roche NeuroToolKit plasma Aβ42/Aβ40 | -0.011 (-0.139 to 0.120) | 0.87 | 0.062 |
| **GFAP** | Roche NeuroToolKit plasma GFAP (ng/mL) | 0.096 (-0.035 to 0.223) | 0.15 | REFERENCE |
|  | Quanterix Neurology 4-Plex plasma GFAP (pg/mL) | 0.076 (-0.059 to 0.209) | 0.27 | 0.98 |

**Supplementary Table 11. Correlations between rates of change plasma biomarker levels and baseline PACC.**Unadjusted Spearman correlations are shown. Correlations between the top performing measure for each analyte and other measures were compared by bootstrapping.

**Full cohort**

| **Analyte** | **Measure** | **Spearman rho** | **p=** | **Comparison** |
| --- | --- | --- | --- | --- |
|  |  | **(95% CI)** |  |  |
| **p-tau217** | Fujirebio Lumipulse plasma p-tau217 (pg/mL) | -0.458 (-0.538 to -0.366) | <0.01 | REFERENCE |
|  | ALZpath Quanterix plasma p-tau217 (pg/mL) | -0.444 (-0.527 to -0.352) | <0.01 | 0.56 |
|  | Janssen LucentAD Quanterix plasma p-tau217 (pg/mL) | -0.442 (-0.527 to -0.350) | <0.01 | 0.54 |
|  | C2N Precivity plasma p-tau217 (pg/mL) | -0.429 (-0.520 to -0.334) | <0.01 | 0.3 |
|  | C2N Precivity plasma %p-tau217 | -0.399 (-0.489 to -0.301) | <0.01 | 0.06 |
| **p-tau181** | Roche NeuroToolKit plasma p-tau181 (pg/mL) | -0.440 (-0.524 to -0.347) | <0.01 | REFERENCE |
|  | Quanterix Neurology 4-Plex plasma p-tau181 (pg/ml) | -0.391 (-0.479 to -0.295) | <0.01 | 0.02 |
| **NfL** | Quanterix Neurology 4-Plex plasma NfL (pg/mL) | -0.354 (-0.449 to -0.254) | <0.01 | REFERENCE |
|  | Roche NeuroToolKit plasma NfL (pg/mL) | -0.333 (-0.422 to -0.240) | <0.01 | 0.53 |
| **GFAP** | Roche NeuroToolKit plasma GFAP (ng/mL) | -0.354 (-0.442 to -0.262) | <0.01 | 1 |
|  | Quanterix Neurology 4-Plex plasma GFAP (pg/mL) | -0.352 (-0.444 to -0.251) | <0.01 | 0.21 |
| **Aβ42/Aβ40** | Fujirebio Lumipulse plasma Aβ42/Aβ40 | 0.281 (0.181 to 0.378) | <0.01 | REFERENCE |
|  | Quanterix Neurology 4-Plex plasma Aβ42/Aβ40 | 0.262 (0.155 to 0.366) | <0.01 | 0.52 |
|  | C2N Precivity plasma Aβ42/Aβ40 | 0.208 (0.108 to 0.303) | <0.01 | 0.13 |
|  | Roche NeuroToolKit plasma Aβ42/Aβ40 | 0.200 (0.096 to 0.297) | <0.01 | 0.09 |

**Amyloid PET positive sub-cohort**

| **Analyte** | **Measure** | **Spearman rho** | **p=** | **Comparison** |
| --- | --- | --- | --- | --- |
|  |  | **(95% CI)** |  |  |
| **p-tau217** | Fujirebio Lumipulse plasma p-tau217 (pg/mL) | -0.502 (-0.627 to -0.355) | <0.01 | REFERENCE |
|  | ALZpath Quanterix plasma p-tau217 (pg/mL) | -0.497 (-0.616 to -0.359) | <0.01 | 0.91 |
|  | Janssen LucentAD Quanterix plasma p-tau217 (pg/mL) | -0.488 (-0.617 to -0.342) | <0.01 | 0.75 |
|  | C2N Precivity plasma %p-tau217 | -0.471 (-0.602 to -0.321) | <0.01 | 0.55 |
|  | C2N Precivity plasma p-tau217 (pg/mL) | -0.460 (-0.590 to -0.311) | <0.01 | 0.35 |
| **p-tau181** | Roche NeuroToolKit plasma p-tau181 (pg/mL) | -0.501 (-0.620 to -0.360) | <0.01 | REFERENCE |
|  | Quanterix Neurology 4-Plex plasma p-tau181 (pg/ml) | -0.447 (-0.582 to -0.289) | <0.01 | 0.29 |
| **Aβ42/Aβ40** | Quanterix Neurology 4-Plex plasma Aβ42/Aβ40 | 0.268 (0.090 to 0.434) | <0.01 | REFERENCE |
|  | Fujirebio Lumipulse plasma Aβ42/Aβ40 | 0.267 (0.097 to 0.424) | <0.01 | 0.32 |
|  | Roche NeuroToolKit plasma Aβ42/Aβ40 | 0.100 (-0.072 to 0.270) | 0.24 | 0.13 |
|  | C2N Precivity plasma Aβ42/Aβ40 | 0.057 (-0.111 to 0.224) | 0.50 | 0.13 |
| **NfL** | Roche NeuroToolKit plasma NfL (pg/mL) | -0.231 (-0.390 to -0.060) | <0.01 | REFERENCE |
|  | Quanterix Neurology 4-Plex plasma NfL (pg/mL) | -0.211 (-0.391 to -0.018) | 0.02 | 0.50 |
| **GFAP** | Roche NeuroToolKit plasma GFAP (ng/mL) | -0.222 (-0.372 to -0.061) | <0.01 | REFERENCE |
|  | Quanterix Neurology 4-Plex plasma GFAP (pg/mL) | -0.197 (-0.365 to -0.018) | 0.04 | 0.23 |

**Amyloid PET negative sub-cohort**

| **Analyte** | **Measure** | **Spearman rho** | **p=** | **Comparison** |
| --- | --- | --- | --- | --- |
|  |  | **(95% CI)** |  |  |
| **NfL** | Quanterix Neurology 4-Plex plasma NfL (pg/mL) | -0.335 (-0.452 to -0.207) | <0.01 | REFERENCE |
|  | Roche NeuroToolKit plasma NfL (pg/mL) | -0.284 (-0.401 to -0.158) | <0.01 | 0.92 |
| **GFAP** | Quanterix Neurology 4-Plex plasma GFAP (pg/mL) | -0.272 (-0.393 to -0.144) | <0.01 | REFERENCE |
|  | Roche NeuroToolKit plasma GFAP (ng/mL) | -0.253 (-0.368 to -0.129) | <0.01 | 0.94 |
| **p-tau181** | Roche NeuroToolKit plasma p-tau181 (pg/mL) | -0.215 (-0.335 to -0.091) | <0.01 | REFERENCE |
|  | Quanterix Neurology 4-Plex plasma p-tau181 (pg/ml) | -0.182 (-0.310 to -0.049) | <0.01 | 0.08 |
| **p-tau217** | Janssen LucentAD Quanterix plasma p-tau217 (pg/mL) | -0.207 (-0.330 to -0.074) | <0.01 | REFERENCE |
|  | Fujirebio Lumipulse plasma p-tau217 (pg/mL) | -0.204 (-0.326 to -0.078) | <0.01 | 0.95 |
|  | ALZpath Quanterix plasma p-tau217 (pg/mL) | -0.194 (-0.317 to -0.061) | <0.01 | 0.73 |
|  | C2N Precivity plasma p-tau217 (pg/mL) | -0.151 (-0.281 to -0.016) | 0.02 | 0.38 |
|  | C2N Precivity plasma %p-tau217 | -0.075 (-0.207 to 0.063) | 0.26 | 0.05 |
| **Aβ42/Aβ40** | C2N Precivity plasma Aβ42/Aβ40 | 0.055 (-0.071 to 0.183) | 0.40 | REFERENCE |
|  | Quanterix Neurology 4-Plex plasma Aβ42/Aβ40 | 0.022 (-0.113 to 0.157) | 0.75 | 0.73 |
|  | Roche NeuroToolKit plasma Aβ42/Aβ40 | -0.016 (-0.148 to 0.117) | 0.81 | 0.53 |
|  | Fujirebio Lumipulse plasma Aβ42/Aβ40 | -0.004 (-0.138 to 0.126) | 0.95 | 0.40 |

**Supplementary Table 12. Correlations between rates of change plasma biomarker levels and rates of change in amyloid PET Centiloid.** Unadjusted Spearman correlations are shown. Correlations between the top performing measure for each analyte and other measures were compared by bootstrapping.

**Full cohort**

| **Analyte** | **Measure** | **Spearman rho** | **p=** | **Comparison** |
| --- | --- | --- | --- | --- |
|  |  | **(95% CI)** |  |  |
| **p-tau217** | ALZpath Quanterix plasma p-tau217 (pg/mL) | 0.332 (0.232 to 0.427) | <0.01 | REFERENCE |
|  | Fujirebio Lumipulse plasma p-tau217 (pg/mL) | 0.316 (0.215 to 0.411) | <0.01 | 0.67 |
|  | Janssen LucentAD Quanterix plasma p-tau217 (pg/mL) | 0.292 (0.186 to 0.393) | <0.01 | 0.29 |
|  | C2N Precivity plasma p-tau217 (pg/mL) | 0.288 (0.183 to 0.389) | <0.01 | 0.31 |
|  | C2N Precivity plasma %p-tau217 | 0.228 (0.125 to 0.326) | <0.01 | 0.054 |
| **p-tau181** | Roche NeuroToolKit plasma p-tau181 (pg/mL) | 0.256 (0.150 to 0.361) | <0.01 | REFERENCE |
|  | Quanterix Neurology 4-Plex plasma p-tau181 (pg/ml) | 0.208 (0.095 to 0.317) | <0.01 | 0.36 |
| **GFAP** | Roche NeuroToolKit plasma GFAP (ng/mL) | 0.194 (0.090 to 0.295) | <0.01 | REFERENCE |
|  | Quanterix Neurology 4-Plex plasma GFAP (pg/mL) | 0.162 (0.049 to 0.275) | <0.01 | 0.96 |
| **Aβ42/Aβ40** | Fujirebio Lumipulse plasma Aβ42/Aβ40 | 0.077 (-0.025 to 0.179) | 0.14 | REFERENCE |
|  | Roche NeuroToolKit plasma Aβ42/Aβ40 | 0.049 (-0.054 to 0.150) | 0.34 | 0.52 |
|  | Quanterix Neurology 4-Plex plasma Aβ42/Aβ40 | 0.034 (-0.075 to 0.144) | 0.54 | 0.59 |
|  | C2N Precivity plasma Aβ42/Aβ40 | 0.017 (-0.085 to 0.118) | 0.74 | 0.28 |
| **NfL** | Quanterix Neurology 4-Plex plasma NfL (pg/mL) | 0.070 (-0.045 to 0.185) | 0.21 | REFERENCE |
|  | Roche NeuroToolKit plasma NfL (pg/mL) | 0.052 (-0.057 to 0.159) | 0.31 | 0.75 |

**Amyloid PET positive sub-cohort**

| **Analyte** | **Measure** | **Spearman rho** | **p=** | **Comparison** |
| --- | --- | --- | --- | --- |
|  |  | **(95% CI)** |  |  |
| **p-tau181** | Quanterix Neurology 4-Plex plasma p-tau181 (pg/ml) | 0.232 (0.051 to 0.401) | 0.01 | REFERENCE |
|  | Roche NeuroToolKit plasma p-tau181 (pg/mL) | 0.196 (0.028 to 0.358) | 0.02 | 0.91 |
| **p-tau217** | Janssen LucentAD Quanterix plasma p-tau217 (pg/mL) | 0.178 (0.003 to 0.346) | 0.04 | REFERENCE |
|  | ALZpath Quanterix plasma p-tau217 (pg/mL) | 0.163 (0.001 to 0.318) | 0.05 | 0.81 |
|  | Fujirebio Lumipulse plasma p-tau217 (pg/mL) | 0.154 (-0.007 to 0.315) | 0.07 | 0.67 |
|  | C2N Precivity plasma %p-tau217 | 0.112 (-0.055 to 0.278) | 0.19 | 0.42 |
|  | C2N Precivity plasma p-tau217 (pg/mL) | 0.074 (-0.094 to 0.242) | 0.38 | 0.18 |
| **GFAP** | Roche NeuroToolKit plasma GFAP (ng/mL) | 0.151 (-0.015 to 0.316) | 0.08 | REFERENCE |
|  | Quanterix Neurology 4-Plex plasma GFAP (pg/mL) | 0.078 (-0.113 to 0.265) | 0.41 | 0.3 |
| **Aβ42/Aβ40** | C2N Precivity plasma Aβ42/Aβ40 | 0.088 (-0.079 to 0.249) | 0.3 | REFERENCE |
|  | Roche NeuroToolKit plasma Aβ42/Aβ40 | 0.086 (-0.078 to 0.247) | 0.32 | 0.87 |
|  | Fujirebio Lumipulse plasma Aβ42/Aβ40 | 0.069 (-0.102 to 0.241) | 0.41 | 0.82 |
|  | Quanterix Neurology 4-Plex plasma Aβ42/Aβ40 | 0.048 (-0.129 to 0.227) | 0.61 | 0.66 |
| **NfL** | Roche NeuroToolKit plasma NfL (pg/mL) | -0.019 (-0.192 to 0.152) | 0.82 | REFERENCE |
|  | Quanterix Neurology 4-Plex plasma NfL (pg/mL) | -0.015 (-0.200 to 0.169) | 0.87 | 0.82 |

**Amyloid PET negative sub-cohort**

| **Analyte** | **Measure** | **Spearman rho** | **p=** | **Comparison** |
| --- | --- | --- | --- | --- |
|  |  | **(95% CI)** |  |  |
| **p-tau217** | ALZpath Quanterix plasma p-tau217 (pg/mL) | 0.363 (0.240 to 0.479) | <0.01 | REFERENCE |
|  | C2N Precivity plasma p-tau217 (pg/mL) | 0.309 (0.176 to 0.433) | <0.01 | 0.42 |
|  | Janssen LucentAD Quanterix plasma p-tau217 (pg/mL) | 0.260 (0.130 to 0.378) | <0.01 | 0.02 |
|  | Fujirebio Lumipulse plasma p-tau217 (pg/mL) | 0.256 (0.121 to 0.386) | <0.01 | 0.029 |
|  | C2N Precivity plasma %p-tau217 | 0.204 (0.067 to 0.333) | <0.01 | 0.057 |
| **p-tau181** | Roche NeuroToolKit plasma p-tau181 (pg/mL) | 0.232 (0.096 to 0.364) | <0.01 | REFERENCE |
|  | Quanterix Neurology 4-Plex plasma p-tau181 (pg/ml) | 0.121 (-0.018 to 0.261) | 0.08 | 0.14 |
| **GFAP** | Roche NeuroToolKit plasma GFAP (ng/mL) | 0.196 (0.062 to 0.325) | <0.01 | REFERENCE |
|  | Quanterix Neurology 4-Plex plasma GFAP (pg/mL) | 0.190 (0.054 to 0.319) | <0.01 | 0.72 |
| **NfL** | Quanterix Neurology 4-Plex plasma NfL (pg/mL) | 0.057 (-0.082 to 0.196) | 0.41 | REFERENCE |
|  | Roche NeuroToolKit plasma NfL (pg/mL) | 0.037 (-0.096 to 0.172) | 0.58 | 0.95 |
| **Aβ42/Aβ40** | Fujirebio Lumipulse plasma Aβ42/Aβ40 | 0.055 (-0.074 to 0.184) | 0.4 | REFERENCE |
|  | Quanterix Neurology 4-Plex plasma Aβ42/Aβ40 | -0.042 (-0.181 to 0.097) | 0.55 | 0.71 |
|  | C2N Precivity plasma Aβ42/Aβ40 | -0.014 (-0.149 to 0.121) | 0.84 | 0.51 |
|  | Roche NeuroToolKit plasma Aβ42/Aβ40 | 0.009 (-0.124 to 0.141) | 0.89 | 0.38 |

**Supplementary Table 13. Correlations between rates of change plasma biomarker levels and rates of change in white matter adjusted amyloid PET SUVR.**Unadjusted Spearman correlations are shown. Correlations between the top performing measure for each analyte and other measures were compared by bootstrapping.

**Full cohort**

| **Analyte** | **Measure** | **Spearman rho** | **p=** | **Comparison** |
| --- | --- | --- | --- | --- |
|  |  | **(95% CI)** |  |  |
| **p-tau217** | ALZpath Quanterix plasma p-tau217 (pg/mL) | 0.354 (0.255 to 0.444) | <0.01 | REFERENCE |
|  | C2N Precivity plasma %p-tau217 | 0.331 (0.234 to 0.420) | <0.01 | 0.66 |
|  | Fujirebio Lumipulse plasma p-tau217 (pg/mL) | 0.318 (0.217 to 0.414) | <0.01 | 0.31 |
|  | C2N Precivity plasma p-tau217 (pg/mL) | 0.301 (0.205 to 0.394) | <0.01 | 0.22 |
|  | Janssen LucentAD Quanterix plasma p-tau217 (pg/mL) | 0.250 (0.146 to 0.347) | <0.01 | 0.0034 |
| **p-tau181** | Roche NeuroToolKit plasma p-tau181 (pg/mL) | 0.209 (0.106 to 0.309) | <0.01 | REFERENCE |
|  | Quanterix Neurology 4-Plex plasma p-tau181 (pg/ml) | 0.180 (0.065 to 0.289) | <0.01 | 0.31 |
| **GFAP** | Roche NeuroToolKit plasma GFAP (ng/mL) | 0.173 (0.069 to 0.274) | <0.01 | REFERENCE |
|  | Quanterix Neurology 4-Plex plasma GFAP (pg/mL) | 0.096 (-0.020 to 0.209) | 0.10 | 0.38 |
| **NfL** | Quanterix Neurology 4-Plex plasma NfL (pg/mL) | 0.093 (-0.022 to 0.207) | 0.11 | REFERENCE |
|  | Roche NeuroToolKit plasma NfL (pg/mL) | 0.092 (-0.013 to 0.195) | 0.09 | 0.60 |
| **Aβ42/Aβ40** | Fujirebio Lumipulse plasma Aβ42/Aβ40 | -0.080 (-0.184 to 0.031) | 0.14 | REFERENCE |
|  | C2N Precivity plasma Aβ42/Aβ40 | -0.044 (-0.150 to 0.066) | 0.42 | 0.52 |
|  | Roche NeuroToolKit plasma Aβ42/Aβ40 | -0.036 (-0.142 to 0.072) | 0.51 | 0.42 |
|  | Quanterix Neurology 4-Plex plasma Aβ42/Aβ40 | -0.025 (-0.136 to 0.088) | 0.67 | 0.31 |

**Amyloid PET positive sub-cohort**

| **Analyte** | **Measure** | **Spearman rho** | **p=** | **Comparison** |
| --- | --- | --- | --- | --- |
|  |  | **(95% CI)** |  |  |
| **p-tau217** | C2N Precivity plasma %p-tau217 | 0.331 (0.155 to 0.483) | <0.01 | REFERENCE |
|  | ALZpath Quanterix plasma p-tau217 (pg/mL) | 0.245 (0.073 to 0.403) | <0.01 | 0.35 |
|  | C2N Precivity plasma p-tau217 (pg/mL) | 0.192 (0.015 to 0.362) | 0.03 | 0.03 |
|  | Janssen LucentAD Quanterix plasma p-tau217 (pg/mL) | 0.173 (-0.007 to 0.344) | 0.05 | 0.05 |
|  | Fujirebio Lumipulse plasma p-tau217 (pg/mL) | 0.132 (-0.052 to 0.305) | 0.14 | 0.02 |
| **p-tau181** | Roche NeuroToolKit plasma p-tau181 (pg/mL) | 0.186 (0.014 to 0.351) | 0.04 | REFERENCE |
|  | Quanterix Neurology 4-Plex plasma p-tau181 (pg/ml) | 0.069 (-0.128 to 0.259) | 0.49 | 0.22 |
| **GFAP** | Roche NeuroToolKit plasma GFAP (ng/mL) | 0.096 (-0.082 to 0.275) | 0.28 | REFERENCE |
|  | Quanterix Neurology 4-Plex plasma GFAP (pg/mL) | -0.005 (-0.204 to 0.195) | 0.96 | 0.61 |
| **Aβ42/Aβ40** | Roche NeuroToolKit plasma Aβ42/Aβ40 | 0.083 (-0.103 to 0.260) | 0.36 | REFERENCE |
|  | Quanterix Neurology 4-Plex plasma Aβ42/Aβ40 | -0.013 (-0.220 to 0.199) | 0.9 | 0.17 |
|  | C2N Precivity plasma Aβ42/Aβ40 | -0.012 (-0.185 to 0.163) | 0.9 | 0.4 |
|  | Fujirebio Lumipulse plasma Aβ42/Aβ40 | -0.001 (-0.190 to 0.183) | 0.99 | 0.34 |
| **NfL** | Roche NeuroToolKit plasma NfL (pg/mL) | 0.082 (-0.097 to 0.255) | 0.36 | REFERENCE |
|  | Quanterix Neurology 4-Plex plasma NfL (pg/mL) | 0.032 (-0.170 to 0.230) | 0.75 | 0.22 |

**Amyloid PET negative sub-cohort**

| **Analyte** | **Measure** | **Spearman rho** | **p=** | **Comparison** |
| --- | --- | --- | --- | --- |
|  |  | **(95% CI)** |  |  |
| **p-tau217** | C2N Precivity plasma %p-tau217 | 0.269 (0.135 to 0.391) | <0.01 | REFERENCE |
|  | ALZpath Quanterix plasma p-tau217 (pg/mL) | 0.267 (0.132 to 0.391) | <0.01 | 0.98 |
|  | Fujirebio Lumipulse plasma p-tau217 (pg/mL) | 0.232 (0.095 to 0.361) | <0.01 | 0.68 |
|  | C2N Precivity plasma p-tau217 (pg/mL) | 0.208 (0.074 to 0.335) | <0.01 | 0.18 |
|  | Janssen LucentAD Quanterix plasma p-tau217 (pg/mL) | 0.159 (0.023 to 0.290) | 0.02 | 0.18 |
| **Aβ42/Aβ40** | Fujirebio Lumipulse plasma Aβ42/Aβ40 | -0.165 (-0.292 to -0.033) | 0.02 | REFERENCE |
|  | Roche NeuroToolKit plasma Aβ42/Aβ40 | -0.133 (-0.263 to -0.002) | 0.05 | 0.7 |
|  | Quanterix Neurology 4-Plex plasma Aβ42/Aβ40 | -0.126 (-0.260 to 0.013) | 0.08 | 0.53 |
|  | C2N Precivity plasma Aβ42/Aβ40 | -0.101 (-0.243 to 0.040) | 0.14 | 0.41 |
| **p-tau181** | Quanterix Neurology 4-Plex plasma p-tau181 (pg/ml) | 0.131 (-0.012 to 0.270) | 0.07 | REFERENCE |
|  | Roche NeuroToolKit plasma p-tau181 (pg/mL) | 0.108 (-0.028 to 0.242) | 0.12 | 0.81 |
| **GFAP** | Roche NeuroToolKit plasma GFAP (ng/mL) | 0.131 (-0.004 to 0.261) | 0.06 | REFERENCE |
|  | Quanterix Neurology 4-Plex plasma GFAP (pg/mL) | 0.050 (-0.092 to 0.192) | 0.49 | 0.62 |
| **NfL** | Quanterix Neurology 4-Plex plasma NfL (pg/mL) | 0.017 (-0.127 to 0.158) | 0.82 | REFERENCE |
|  | Roche NeuroToolKit plasma NfL (pg/mL) | -0.006 (-0.137 to 0.122) | 0.93 | 0.58 |

**Supplementary Table 14. Correlations between rates of change plasma biomarker levels and rates of change in composite reference adjusted amyloid PET SUVR.** Unadjusted Spearman correlations are shown. Correlations between the top performing measure for each analyte and other measures were compared by bootstrapping.

**Full cohort**

| **Analyte** | **Measure** | **Spearman rho** | **p=** | **Comparison** |
| --- | --- | --- | --- | --- |
|  |  | **(95% CI)** |  |  |
| **p-tau217** | ALZpath Quanterix plasma p-tau217 (pg/mL) | 0.417 (0.322 to 0.504) | <0.01 | REFERENCE |
|  | Fujirebio Lumipulse plasma p-tau217 (pg/mL) | 0.402 (0.310 to 0.490) | <0.01 | 0.68 |
|  | C2N Precivity plasma p-tau217 (pg/mL) | 0.357 (0.262 to 0.445) | <0.01 | 0.13 |
|  | Janssen LucentAD Quanterix plasma p-tau217 (pg/mL) | 0.333 (0.234 to 0.429) | <0.01 | 0.028 |
|  | C2N Precivity plasma %p-tau217 | 0.311 (0.216 to 0.404) | <0.01 | 0.047 |
| **p-tau181** | Roche NeuroToolKit plasma p-tau181 (pg/mL) | 0.289 (0.190 to 0.382) | <0.01 | REFERENCE |
|  | Quanterix Neurology 4-Plex plasma p-tau181 (pg/ml) | 0.254 (0.147 to 0.358) | <0.01 | 0.25 |
| **GFAP** | Roche NeuroToolKit plasma GFAP (ng/mL) | 0.176 (0.074 to 0.275) | <0.01 | REFERENCE |
|  | Quanterix Neurology 4-Plex plasma GFAP (pg/mL) | 0.107 (-0.005 to 0.216) | 0.06 | 0.55 |
| **NfL** | Roche NeuroToolKit plasma NfL (pg/mL) | 0.119 (0.018 to 0.220) | 0.02 | REFERENCE |
|  | Quanterix Neurology 4-Plex plasma NfL (pg/mL) | 0.116 (0.004 to 0.223) | 0.04 | 0.54 |
| **Aβ42/Aβ40** | Quanterix Neurology 4-Plex plasma Aβ42/Aβ40 | 0.017 (-0.093 to 0.127) | 0.76 | REFERENCE |
|  | Roche NeuroToolKit plasma Aβ42/Aβ40 | 0.011 (-0.090 to 0.112) | 0.83 | 0.82 |
|  | Fujirebio Lumipulse plasma Aβ42/Aβ40 | 0.007 (-0.094 to 0.109) | 0.89 | 0.76 |
|  | C2N Precivity plasma Aβ42/Aβ40 | -0.002 (-0.107 to 0.101) | 0.97 | 0.89 |

**Amyloid PET positive sub-cohort**

| **Analyte** | **Measure** | **Spearman rho** | **p=** | **Comparison** |
| --- | --- | --- | --- | --- |
|  |  | **(95% CI)** |  |  |
| **p-tau217** | ALZpath Quanterix plasma p-tau217 (pg/mL) | 0.223 (0.056 to 0.378) | <0.01 | REFERENCE |
|  | C2N Precivity plasma %p-tau217 | 0.187 (0.017 to 0.346) | 0.03 | 0.67 |
|  | Janssen LucentAD Quanterix plasma p-tau217 (pg/mL) | 0.179 (0.000 to 0.348) | 0.03 | 0.47 |
|  | Fujirebio Lumipulse plasma p-tau217 (pg/mL) | 0.161 (-0.007 to 0.326) | 0.06 | 0.30 |
|  | C2N Precivity plasma p-tau217 (pg/mL) | 0.114 (-0.055 to 0.278) | 0.18 | 0.10 |
| **p-tau181** | Quanterix Neurology 4-Plex plasma p-tau181 (pg/ml) | 0.214 (0.031 to 0.385) | 0.02 | REFERENCE |
|  | Roche NeuroToolKit plasma p-tau181 (pg/mL) | 0.198 (0.028 to 0.356) | 0.02 | 0.95 |
| **GFAP** | Roche NeuroToolKit plasma GFAP (ng/mL) | 0.135 (-0.033 to 0.305) | 0.11 | REFERENCE |
|  | Quanterix Neurology 4-Plex plasma GFAP (pg/mL) | 0.033 (-0.151 to 0.219) | 0.73 | 0.26 |
| **Aβ42/Aβ40** | Quanterix Neurology 4-Plex plasma Aβ42/Aβ40 | 0.098 (-0.094 to 0.280) | 0.30 | REFERENCE |
|  | Roche NeuroToolKit plasma Aβ42/Aβ40 | 0.085 (-0.082 to 0.247) | 0.32 | 0.84 |
|  | C2N Precivity plasma Aβ42/Aβ40 | 0.077 (-0.083 to 0.233) | 0.36 | 0.93 |
|  | Fujirebio Lumipulse plasma Aβ42/Aβ40 | 0.048 (-0.125 to 0.216) | 0.57 | 0.58 |
| **NfL** | Roche NeuroToolKit plasma NfL (pg/mL) | 0.054 (-0.114 to 0.217) | 0.53 | REFERENCE |
|  | Quanterix Neurology 4-Plex plasma NfL (pg/mL) | 0.011 (-0.169 to 0.194) | 0.91 | 0.34 |

**Amyloid PET negative sub-cohort**

| **Analyte** | **Measure** | **Spearman rho** | **p=** | **Comparison** |
| --- | --- | --- | --- | --- |
|  |  | **(95% CI)** |  |  |
| **p-tau217** | ALZpath Quanterix plasma p-tau217 (pg/mL) | 0.390 (0.266 to 0.504) | <0.01 | REFERENCE |
|  | Fujirebio Lumipulse plasma p-tau217 (pg/mL) | 0.317 (0.190 to 0.435) | <0.01 | 0.17 |
|  | C2N Precivity plasma p-tau217 (pg/mL) | 0.316 (0.190 to 0.435) | <0.01 | 0.25 |
|  | C2N Precivity plasma %p-tau217 | 0.286 (0.155 to 0.408) | <0.01 | 0.18 |
|  | Janssen LucentAD Quanterix plasma p-tau217 (pg/mL) | 0.267 (0.139 to 0.391) | <0.01 | 0.01 |
| **p-tau181** | Roche NeuroToolKit plasma p-tau181 (pg/mL) | 0.220 (0.091 to 0.344) | <0.01 | REFERENCE |
|  | Quanterix Neurology 4-Plex plasma p-tau181 (pg/ml) | 0.163 (0.026 to 0.297) | 0.02 | 0.17 |
| **GFAP** | Roche NeuroToolKit plasma GFAP (ng/mL) | 0.123 (-0.006 to 0.249) | 0.06 | REFERENCE |
|  | Quanterix Neurology 4-Plex plasma GFAP (pg/mL) | 0.059 (-0.073 to 0.193) | 0.39 | 0.74 |
| **Aβ42/Aβ40** | Quanterix Neurology 4-Plex plasma Aβ42/Aβ40 | -0.119 (-0.245 to 0.018) | 0.09 | REFERENCE |
|  | Fujirebio Lumipulse plasma Aβ42/Aβ40 | -0.093 (-0.216 to 0.032) | 0.16 | 0.88 |
|  | Roche NeuroToolKit plasma Aβ42/Aβ40 | -0.091 (-0.215 to 0.039) | 0.17 | 0.94 |
|  | C2N Precivity plasma Aβ42/Aβ40 | -0.078 (-0.210 to 0.057) | 0.24 | 0.81 |
| **NfL** | Quanterix Neurology 4-Plex plasma NfL (pg/mL) | 0.054 (-0.085 to 0.191) | 0.44 | REFERENCE |
|  | Roche NeuroToolKit plasma NfL (pg/mL) | 0.052 (-0.074 to 0.179) | 0.43 | 0.92 |

**Supplementary Table 15. Correlations between rates of change plasma biomarker levels and rates of change in early tau PET.** Unadjusted Spearman correlations are shown. Correlations between the top performing measure for each analyte and other measures were compared by bootstrapping.

**Full cohort**

| **Analyte** | **Measure** | **Spearman rho** | **p=** | **Comparison** |
| --- | --- | --- | --- | --- |
|  |  | **(95% CI)** |  |  |
| **Aβ42/Aβ40** | Fujirebio Lumipulse plasma Aβ42/Aβ40 | 0.195 (-0.012 to 0.397) | 0.06 | REFERENCE |
|  | Quanterix Neurology 4-Plex plasma Aβ42/Aβ40 | 0.112 (-0.129 to 0.340) | 0.36 | 0.91 |
|  | C2N Precivity plasma Aβ42/Aβ40 | -0.049 (-0.244 to 0.147) | 0.65 | 0.22 |
|  | Roche NeuroToolKit plasma Aβ42/Aβ40 | 0.014 (-0.198 to 0.220) | 0.89 | 0.11 |
| **GFAP** | Roche NeuroToolKit plasma GFAP (ng/mL) | 0.099 (-0.120 to 0.317) | 0.35 | REFERENCE |
|  | Quanterix Neurology 4-Plex plasma GFAP (pg/mL) | -0.016 (-0.257 to 0.228) | 0.89 | 0.64 |
| **p-tau181** | Roche NeuroToolKit plasma p-tau181 (pg/mL) | 0.087 (-0.120 to 0.291) | 0.41 | REFERENCE |
|  | Quanterix Neurology 4-Plex plasma p-tau181 (pg/ml) | -0.019 (-0.256 to 0.231) | 0.87 | 0.51 |
| **p-tau217** | Fujirebio Lumipulse plasma p-tau217 (pg/mL) | 0.077 (-0.145 to 0.292) | 0.47 | REFERENCE |
|  | Janssen LucentAD Quanterix plasma p-tau217 (pg/mL) | 0.031 (-0.182 to 0.249) | 0.77 | 0.56 |
|  | ALZpath Quanterix plasma p-tau217 (pg/mL) | 0.031 (-0.187 to 0.252) | 0.77 | 0.5 |
|  | C2N Precivity plasma p-tau217 (pg/mL) | 0.019 (-0.191 to 0.231) | 0.86 | 0.44 |
|  | C2N Precivity plasma %p-tau217 | -0.010 (-0.208 to 0.197) | 0.92 | 0.45 |
| **NfL** | Quanterix Neurology 4-Plex plasma NfL (pg/mL) | -0.048 (-0.299 to 0.206) | 0.69 | REFERENCE |
|  | Roche NeuroToolKit plasma NfL (pg/mL) | -0.045 (-0.260 to 0.178) | 0.67 | 0.56 |

**Amyloid PET positive sub-cohort**

| **Analyte** | **Measure** | **Spearman rho**  **(95% CI)** | **p=** | **Comparison** |
| --- | --- | --- | --- | --- |
| **NfL** | Roche NeuroToolKit plasma NfL (pg/mL) | -0.336 (-0.608 to -0.007) | 0.04 | REFERENCE |
|  | Quanterix Neurology 4-Plex plasma NfL (pg/mL) | -0.230 (-0.623 to 0.230) | 0.27 | 0.44 |
| **GFAP** | Quanterix Neurology 4-Plex plasma GFAP (pg/mL) | -0.308 (-0.658 to 0.141) | 0.13 | REFERENCE |
|  | Roche NeuroToolKit plasma GFAP (ng/mL) | -0.034 (-0.374 to 0.316) | 0.84 | 0.29 |
| **Aβ42/Aβ40** | Fujirebio Lumipulse plasma Aβ42/Aβ40 | 0.284 (-0.052 to 0.569) | 0.08 | REFERENCE |
|  | Roche NeuroToolKit plasma Aβ42/Aβ40 | 0.063 (-0.246 to 0.365) | 0.70 | 0.2 |
|  | C2N Precivity plasma Aβ42/Aβ40 | 0.056 (-0.259 to 0.359) | 0.73 | 0.2 |
|  | Quanterix Neurology 4-Plex plasma Aβ42/Aβ40 | 0.019 (-0.378 to 0.428) | 0.93 | 0.66 |
| **p-tau217** | Janssen LucentAD Quanterix plasma p-tau217 (pg/mL) | -0.138 (-0.466 to 0.208) | 0.40 | REFERENCE |
|  | ALZpath Quanterix plasma p-tau217 (pg/mL) | -0.101 (-0.447 to 0.271) | 0.54 | 0.77 |
|  | Fujirebio Lumipulse plasma p-tau217 (pg/mL) | 0.098 (-0.246 to 0.435) | 0.55 | 0.85 |
|  | C2N Precivity plasma p-tau217 (pg/mL) | -0.046 (-0.388 to 0.299) | 0.78 | 0.48 |
|  | C2N Precivity plasma %p-tau217 | -0.040 (-0.353 to 0.287) | 0.81 | 0.41 |
| **p-tau181** | Roche NeuroToolKit plasma p-tau181 (pg/mL) | -0.061 (-0.397 to 0.272) | 0.71 | REFERENCE |
|  | Quanterix Neurology 4-Plex plasma p-tau181 (pg/ml) | -0.044 (-0.450 to 0.375) | 0.84 | 0.85 |

**Amyloid PET negative sub-cohort**

| **Analyte** | **Measure** | **Spearman rho** | **p=** | **Comparison** |
| --- | --- | --- | --- | --- |
|  |  | **(95% CI)** |  |  |
| **Aβ42/Aβ40** | Quanterix Neurology 4-Plex plasma Aβ42/Aβ40 | 0.209 (-0.097 to 0.480) | 0.17 | REFERENCE |
|  | C2N Precivity plasma Aβ42/Aβ40 | -0.151 (-0.397 to 0.115) | 0.28 | 0.64 |
|  | Fujirebio Lumipulse plasma Aβ42/Aβ40 | 0.113 (-0.173 to 0.394) | 0.42 | 0.42 |
|  | Roche NeuroToolKit plasma Aβ42/Aβ40 | -0.018 (-0.298 to 0.255) | 0.90 | 0.21 |
| **NfL** | Roche NeuroToolKit plasma NfL (pg/mL) | 0.170 (-0.131 to 0.447) | 0.23 | REFERENCE |
|  | Quanterix Neurology 4-Plex plasma NfL (pg/mL) | 0.063 (-0.267 to 0.374) | 0.68 | 0.36 |
| **GFAP** | Roche NeuroToolKit plasma GFAP (ng/mL) | 0.169 (-0.113 to 0.441) | 0.23 | REFERENCE |
|  | Quanterix Neurology 4-Plex plasma GFAP (pg/mL) | 0.139 (-0.163 to 0.421) | 0.37 | 0.98 |
| **p-tau181** | Roche NeuroToolKit plasma p-tau181 (pg/mL) | 0.158 (-0.112 to 0.417) | 0.26 | REFERENCE |
|  | Quanterix Neurology 4-Plex plasma p-tau181 (pg/ml) | -0.005 (-0.324 to 0.311) | 0.97 | 0.27 |
| **p-tau217** | Janssen LucentAD Quanterix plasma p-tau217 (pg/mL) | 0.135 (-0.159 to 0.409) | 0.34 | REFERENCE |
|  | ALZpath Quanterix plasma p-tau217 (pg/mL) | 0.080 (-0.195 to 0.349) | 0.57 | 0.56 |
|  | C2N Precivity plasma p-tau217 (pg/mL) | -0.055 (-0.328 to 0.230) | 0.70 | 0.6 |
|  | Fujirebio Lumipulse plasma p-tau217 (pg/mL) | -0.051 (-0.338 to 0.251) | 0.72 | 0.61 |
|  | C2N Precivity plasma %p-tau217 | -0.044 (-0.310 to 0.230) | 0.75 | 0.54 |

**Supplementary Table 16. Correlations between rates of change plasma biomarker levels and rates of change in late tau PET.** Unadjusted Spearman correlations are shown. Correlations between the top performing measure for each analyte and other measures were compared by bootstrapping.

**Full cohort**

| **Analyte** | **Measure** | **Spearman rho** | **p=** | **Comparison** |
| --- | --- | --- | --- | --- |
|  |  | **(95% CI)** |  |  |
| **p-tau217** | Fujirebio Lumipulse plasma p-tau217 (pg/mL) | 0.165 (-0.056 to 0.373) | 0.12 | REFERENCE |
|  | ALZpath Quanterix plasma p-tau217 (pg/mL) | 0.155 (-0.066 to 0.362) | 0.14 | 0.9 |
|  | Janssen LucentAD Quanterix plasma p-tau217 (pg/mL) | 0.135 (-0.086 to 0.341) | 0.20 | 0.73 |
|  | C2N Precivity plasma p-tau217 (pg/mL) | 0.029 (-0.193 to 0.246) | 0.79 | 0.17 |
|  | C2N Precivity plasma %p-tau217 | -0.016 (-0.235 to 0.199) | 0.88 | 0.22 |
| **Aβ42/Aβ40** | C2N Precivity plasma Aβ42/Aβ40 | -0.160 (-0.357 to 0.045) | 0.13 | REFERENCE |
|  | Fujirebio Lumipulse plasma Aβ42/Aβ40 | 0.091 (-0.124 to 0.297) | 0.39 | 0.58 |
|  | Quanterix Neurology 4-Plex plasma Aβ42/Aβ40 | 0.052 (-0.187 to 0.288) | 0.67 | 0.3 |
|  | Roche NeuroToolKit plasma Aβ42/Aβ40 | -0.047 (-0.253 to 0.160) | 0.66 | 0.25 |
| **p-tau181** | Roche NeuroToolKit plasma p-tau181 (pg/mL) | 0.094 (-0.119 to 0.296) | 0.38 | REFERENCE |
|  | Quanterix Neurology 4-Plex plasma p-tau181 (pg/ml) | 0.090 (-0.157 to 0.330) | 0.46 | 0.93 |
| **GFAP** | Roche NeuroToolKit plasma GFAP (ng/mL) | 0.052 (-0.173 to 0.265) | 0.62 | REFERENCE |
|  | Quanterix Neurology 4-Plex plasma GFAP (pg/mL) | -0.008 (-0.258 to 0.240) | 0.95 | 0.63 |
| **NfL** | Quanterix Neurology 4-Plex plasma NfL (pg/mL) | -0.004 (-0.257 to 0.246) | 0.97 | REFERENCE |
|  | Roche NeuroToolKit plasma NfL (pg/mL) | 0.004 (-0.216 to 0.224) | 0.97 | 0.94 |

**Amyloid PET positive sub-cohort**

| **Analyte** | **Measure** | **Spearman rho**  **(95% CI)** | **p=** | **Comparison** |
| --- | --- | --- | --- | --- |
| **p-tau217** | Fujirebio Lumipulse plasma p-tau217 (pg/mL) | 0.277 (-0.073 to 0.577) | 0.09 | REFERENCE |
|  | ALZpath Quanterix plasma p-tau217 (pg/mL) | 0.270 (-0.092 to 0.580) | 0.10 | 0.96 |
|  | Janssen LucentAD Quanterix plasma p-tau217 (pg/mL) | 0.225 (-0.129 to 0.543) | 0.17 | 0.65 |
|  | C2N Precivity plasma p-tau217 (pg/mL) | 0.136 (-0.235 to 0.479) | 0.41 | 0.17 |
|  | C2N Precivity plasma %p-tau217 | 0.025 (-0.327 to 0.356) | 0.88 | 0.13 |
| **NfL** | Roche NeuroToolKit plasma NfL (pg/mL) | -0.161 (-0.456 to 0.162) | 0.33 | REFERENCE |
|  | Quanterix Neurology 4-Plex plasma NfL (pg/mL) | -0.004 (-0.419 to 0.417) | 0.99 | 0.67 |
| **p-tau181** | Quanterix Neurology 4-Plex plasma p-tau181 (pg/ml) | 0.153 (-0.264 to 0.554) | 0.46 | REFERENCE |
|  | Roche NeuroToolKit plasma p-tau181 (pg/mL) | 0.064 (-0.284 to 0.403) | 0.70 | 0.72 |
| **GFAP** | Roche NeuroToolKit plasma GFAP (ng/mL) | 0.101 (-0.266 to 0.438) | 0.54 | REFERENCE |
|  | Quanterix Neurology 4-Plex plasma GFAP (pg/mL) | -0.007 (-0.458 to 0.475) | 0.98 | 0.37 |
| **Aβ42/Aβ40** | C2N Precivity plasma Aβ42/Aβ40 | -0.084 (-0.377 to 0.227) | 0.61 | REFERENCE |
|  | Quanterix Neurology 4-Plex plasma Aβ42/Aβ40 | -0.066 (-0.459 to 0.343) | 0.75 | 0.68 |
|  | Fujirebio Lumipulse plasma Aβ42/Aβ40 | 0.062 (-0.254 to 0.367) | 0.71 | 0.86 |
|  | Roche NeuroToolKit plasma Aβ42/Aβ40 | -0.044 (-0.340 to 0.254) | 0.79 | 0.69 |

**Amyloid PET negative sub-cohort**

| **Analyte** | **Measure** | **Spearman rho** | **p=** | **Comparison** |
| --- | --- | --- | --- | --- |
|  |  | **(95% CI)** |  |  |
| **Aβ42/Aβ40** | C2N Precivity plasma Aβ42/Aβ40 | -0.291 (-0.506 to -0.039) | 0.04 | REFERENCE |
|  | Fujirebio Lumipulse plasma Aβ42/Aβ40 | 0.126 (-0.166 to 0.399) | 0.37 | 0.33 |
|  | Roche NeuroToolKit plasma Aβ42/Aβ40 | -0.082 (-0.369 to 0.205) | 0.56 | 0.13 |
|  | Quanterix Neurology 4-Plex plasma Aβ42/Aβ40 | 0.077 (-0.235 to 0.376) | 0.62 | 0.19 |
| **p-tau217** | C2N Precivity plasma p-tau217 (pg/mL) | -0.252 (-0.503 to 0.041) | 0.07 | REFERENCE |
|  | Fujirebio Lumipulse plasma p-tau217 (pg/mL) | -0.156 (-0.442 to 0.146) | 0.27 | 0.48 |
|  | C2N Precivity plasma %p-tau217 | -0.150 (-0.415 to 0.136) | 0.29 | 0.21 |
|  | ALZpath Quanterix plasma p-tau217 (pg/mL) | -0.148 (-0.415 to 0.143) | 0.29 | 0.39 |
|  | Janssen LucentAD Quanterix plasma p-tau217 (pg/mL) | -0.070 (-0.350 to 0.215) | 0.62 | 0.16 |
| **NfL** | Roche NeuroToolKit plasma NfL (pg/mL) | 0.059 (-0.251 to 0.353) | 0.67 | REFERENCE |
|  | Quanterix Neurology 4-Plex plasma NfL (pg/mL) | 0.003 (-0.336 to 0.325) | 0.98 | 0.98 |
| **GFAP** | Roche NeuroToolKit plasma GFAP (ng/mL) | -0.058 (-0.326 to 0.218) | 0.68 | REFERENCE |
|  | Quanterix Neurology 4-Plex plasma GFAP (pg/mL) | -0.039 (-0.336 to 0.248) | 0.80 | 0.62 |
| **p-tau181** | Quanterix Neurology 4-Plex plasma p-tau181 (pg/ml) | -0.048 (-0.343 to 0.251) | 0.76 | REFERENCE |
|  | Roche NeuroToolKit plasma p-tau181 (pg/mL) | -0.038 (-0.298 to 0.224) | 0.79 | 1 |

**Supplementary Table 17. Correlations between rates of change plasma biomarker levels and rates of change in cortical thickness.** Unadjusted Spearman correlations are shown. Correlations between the top performing measure for each analyte and other measures were compared by bootstrapping.

**Full cohort**

| **Analyte** | **Measure** | **Spearman rho** | **p=** | **Comparison** |
| --- | --- | --- | --- | --- |
|  |  | **(95% CI)** |  |  |
| **p-tau217** | Fujirebio Lumipulse plasma p-tau217 (pg/mL) | -0.105 (-0.218 to 0.011) | 0.06 | REFERENCE |
|  | C2N Precivity plasma p-tau217 (pg/mL) | -0.104 (-0.220 to 0.015) | 0.07 | 0.99 |
|  | Janssen LucentAD Quanterix plasma p-tau217 (pg/mL) | -0.075 (-0.188 to 0.041) | 0.18 | 0.52 |
|  | C2N Precivity plasma %p-tau217 | -0.045 (-0.162 to 0.072) | 0.42 | 0.34 |
|  | ALZpath Quanterix plasma p-tau217 (pg/mL) | -0.023 (-0.139 to 0.092) | 0.68 | 0.12 |
| **GFAP** | Roche NeuroToolKit plasma GFAP (ng/mL) | -0.099 (-0.213 to 0.019) | 0.08 | REFERENCE |
|  | Quanterix Neurology 4-Plex plasma GFAP (pg/mL) | -0.082 (-0.206 to 0.043) | 0.21 | 0.51 |
| **NfL** | Quanterix Neurology 4-Plex plasma NfL (pg/mL) | -0.082 (-0.206 to 0.043) | 0.18 | REFERENCE |
|  | Roche NeuroToolKit plasma NfL (pg/mL) | -0.081 (-0.198 to 0.037) | 0.15 | 0.23 |
| **Aβ42/Aβ40** | Quanterix Neurology 4-Plex plasma Aβ42/Aβ40 | -0.079 (-0.201 to 0.046) | 0.2 | REFERENCE |
|  | Fujirebio Lumipulse plasma Aβ42/Aβ40 | 0.052 (-0.064 to 0.163) | 0.36 | 0.76 |
|  | C2N Precivity plasma Aβ42/Aβ40 | 0.029 (-0.084 to 0.141) | 0.6 | 0.63 |
|  | Roche NeuroToolKit plasma Aβ42/Aβ40 | -0.025 (-0.138 to 0.089) | 0.66 | 0.27 |
| **p-tau181** | Roche NeuroToolKit plasma p-tau181 (pg/mL) | -0.064 (-0.174 to 0.049) | 0.26 | REFERENCE |
|  | Quanterix Neurology 4-Plex plasma p-tau181 (pg/ml) | -0.010 (-0.131 to 0.111) | 0.87 | 0.48 |

**Amyloid PET positive sub-cohort**

| **Analyte** | **Measure** | **Spearman rho** | **p=** | **Comparison** |
| --- | --- | --- | --- | --- |
|  |  | **(95% CI)** |  |  |
| **NfL** | Quanterix Neurology 4-Plex plasma NfL (pg/mL) | -0.158 (-0.359 to 0.057) | 0.14 | REFERENCE |
|  | Roche NeuroToolKit plasma NfL (pg/mL) | -0.113 (-0.298 to 0.083) | 0.24 | 0.26 |
| **p-tau217** | Janssen LucentAD Quanterix plasma p-tau217 (pg/mL) | -0.122 (-0.304 to 0.069) | 0.20 | REFERENCE |
|  | Fujirebio Lumipulse plasma p-tau217 (pg/mL) | -0.116 (-0.305 to 0.074) | 0.22 | 0.93 |
|  | C2N Precivity plasma p-tau217 (pg/mL) | -0.069 (-0.264 to 0.132) | 0.47 | 0.5 |
|  | ALZpath Quanterix plasma p-tau217 (pg/mL) | 0.016 (-0.165 to 0.198) | 0.87 | 0.31 |
|  | C2N Precivity plasma %p-tau217 | -0.010 (-0.199 to 0.184) | 0.92 | 0.23 |
| **Aβ42/Aβ40** | Roche NeuroToolKit plasma Aβ42/Aβ40 | -0.080 (-0.282 to 0.124) | 0.4 | REFERENCE |
|  | Fujirebio Lumipulse plasma Aβ42/Aβ40 | -0.066 (-0.260 to 0.134) | 0.49 | 0.86 |
|  | Quanterix Neurology 4-Plex plasma Aβ42/Aβ40 | -0.063 (-0.280 to 0.151) | 0.56 | 0.9 |
|  | C2N Precivity plasma Aβ42/Aβ40 | -0.007 (-0.197 to 0.181) | 0.94 | 0.39 |
| **p-tau181** | Quanterix Neurology 4-Plex plasma p-tau181 (pg/ml) | -0.059 (-0.267 to 0.152) | 0.58 | REFERENCE |
|  | Roche NeuroToolKit plasma p-tau181 (pg/mL) | -0.032 (-0.212 to 0.151) | 0.74 | 0.93 |
| **GFAP** | Roche NeuroToolKit plasma GFAP (ng/mL) | 0.025 (-0.161 to 0.219) | 0.79 | REFERENCE |
|  | Quanterix Neurology 4-Plex plasma GFAP (pg/mL) | 0.019 (-0.200 to 0.228) | 0.86 | 0.33 |

**Amyloid PET negative sub-cohort**

| **Analyte** | **Measure** | **Spearman rho** | **p=** | **Comparison** |
| --- | --- | --- | --- | --- |
|  |  | **(95% CI)** |  |  |
| **Aβ42/Aβ40** | Fujirebio Lumipulse plasma Aβ42/Aβ40 | 0.158 (0.023 to 0.291) | 0.03 | REFERENCE |
|  | C2N Precivity plasma Aβ42/Aβ40 | 0.075 (-0.066 to 0.211) | 0.29 | 0.3 |
|  | Quanterix Neurology 4-Plex plasma Aβ42/Aβ40 | -0.049 (-0.201 to 0.106) | 0.51 | 0.23 |
|  | Roche NeuroToolKit plasma Aβ42/Aβ40 | 0.027 (-0.110 to 0.162) | 0.70 | 0.069 |
| **GFAP** | Roche NeuroToolKit plasma GFAP (ng/mL) | -0.138 (-0.281 to 0.006) | 0.05 | REFERENCE |
|  | Quanterix Neurology 4-Plex plasma GFAP (pg/mL) | -0.099 (-0.242 to 0.047) | 0.18 | 0.9 |
| **p-tau181** | Quanterix Neurology 4-Plex plasma p-tau181 (pg/ml) | 0.097 (-0.055 to 0.246) | 0.20 | REFERENCE |
|  | Roche NeuroToolKit plasma p-tau181 (pg/mL) | -0.027 (-0.165 to 0.115) | 0.71 | 0.61 |
| **p-tau217** | C2N Precivity plasma p-tau217 (pg/mL) | -0.042 (-0.189 to 0.099) | 0.56 | REFERENCE |
|  | Fujirebio Lumipulse plasma p-tau217 (pg/mL) | 0.029 (-0.117 to 0.174) | 0.68 | 0.84 |
|  | Janssen LucentAD Quanterix plasma p-tau217 (pg/mL) | 0.022 (-0.123 to 0.166) | 0.76 | 0.75 |
|  | C2N Precivity plasma %p-tau217 | -0.020 (-0.163 to 0.124) | 0.77 | 0.65 |
|  | ALZpath Quanterix plasma p-tau217 (pg/mL) | 0.013 (-0.133 to 0.158) | 0.85 | 0.63 |
| **NfL** | Roche NeuroToolKit plasma NfL (pg/mL) | -0.041 (-0.187 to 0.108) | 0.57 | REFERENCE |
|  | Quanterix Neurology 4-Plex plasma NfL (pg/mL) | 0.002 (-0.152 to 0.151) | 0.98 | 0.41 |

**Supplementary Table 18. Correlations between rates of change plasma biomarker levels and rates of change in CDR-SB.** Unadjusted Spearman correlations are shown. Correlations between the top performing measure for each analyte and other measures were compared by bootstrapping.

**Full cohort**

| **Analyte** | **Measure** | **Spearman rho** | **p=** | **Comparison** |
| --- | --- | --- | --- | --- |
|  |  | **(95% CI)** |  |  |
| **NfL** | Quanterix Neurology 4-Plex plasma NfL (pg/mL) | 0.191 (0.074 to 0.301) | <0.01 | REFERENCE |
|  | Roche NeuroToolKit plasma NfL (pg/mL) | 0.177 (0.069 to 0.280) | <0.01 | 0.18 |
| **Aβ42/Aβ40** | Fujirebio Lumipulse plasma Aβ42/Aβ40 | 0.130 (0.023 to 0.234) | <0.01 | REFERENCE |
|  | Quanterix Neurology 4-Plex plasma Aβ42/Aβ40 | 0.130 (0.022 to 0.233) | 0.02 | 0.63 |
|  | C2N Precivity plasma Aβ42/Aβ40 | 0.097 (-0.010 to 0.200) | 0.06 | 0.51 |
|  | Roche NeuroToolKit plasma Aβ42/Aβ40 | 0.064 (-0.041 to 0.168) | 0.22 | 0.15 |
| **p-tau217** | C2N Precivity plasma %p-tau217 | 0.135 (0.027 to 0.242) | 0.01 | REFERENCE |
|  | Fujirebio Lumipulse plasma p-tau217 (pg/mL) | 0.130 (0.019 to 0.240) | 0.01 | 0.94 |
|  | C2N Precivity plasma p-tau217 (pg/mL) | 0.130 (0.016 to 0.243) | 0.01 | 0.9 |
|  | Janssen LucentAD Quanterix plasma p-tau217 (pg/mL) | 0.100 (-0.010 to 0.207) | 0.05 | 0.53 |
|  | ALZpath Quanterix plasma p-tau217 (pg/mL) | 0.040 (-0.072 to 0.151) | 0.44 | 0.087 |
| **GFAP** | Quanterix Neurology 4-Plex plasma GFAP (pg/mL) | 0.058 (-0.052 to 0.168) | 0.30 | REFERENCE |
|  | Roche NeuroToolKit plasma GFAP (ng/mL) | 0.010 (-0.095 to 0.114) | 0.85 | 0.41 |
| **p-tau181** | Roche NeuroToolKit plasma p-tau181 (pg/mL) | 0.043 (-0.063 to 0.151) | 0.41 | REFERENCE |
|  | Quanterix Neurology 4-Plex plasma p-tau181 (pg/ml) | 0.035 (-0.074 to 0.141) | 0.53 | 0.57 |

**Amyloid PET positive sub-cohort**

| **Analyte** | **Measure** | **Spearman rho** | **p=** | **Comparison** |
| --- | --- | --- | --- | --- |
|  |  | **(95% CI)** |  |  |
| **NfL** | Quanterix Neurology 4-Plex plasma NfL (pg/mL) | 0.230 (0.051 to 0.399) | 0.01 | REFERENCE |
|  | Roche NeuroToolKit plasma NfL (pg/mL) | 0.217 (0.053 to 0.375) | 0.01 | 0.5 |
| **Aβ42/Aβ40** | Quanterix Neurology 4-Plex plasma Aβ42/Aβ40 | 0.167 (-0.018 to 0.343) | 0.08 | REFERENCE |
|  | Fujirebio Lumipulse plasma Aβ42/Aβ40 | 0.150 (-0.023 to 0.318) | 0.08 | 0.87 |
|  | Roche NeuroToolKit plasma Aβ42/Aβ40 | 0.088 (-0.086 to 0.263) | 0.3 | 0.69 |
|  | C2N Precivity plasma Aβ42/Aβ40 | 0.034 (-0.149 to 0.212) | 0.69 | 0.38 |
| **p-tau217** | Fujirebio Lumipulse plasma p-tau217 (pg/mL) | 0.149 (-0.025 to 0.313) | 0.08 | REFERENCE |
|  | C2N Precivity plasma p-tau217 (pg/mL) | 0.135 (-0.053 to 0.311) | 0.11 | 0.79 |
|  | C2N Precivity plasma %p-tau217 | 0.102 (-0.079 to 0.280) | 0.23 | 0.56 |
|  | Janssen LucentAD Quanterix plasma p-tau217 (pg/mL) | 0.056 (-0.118 to 0.227) | 0.51 | 0.17 |
|  | ALZpath Quanterix plasma p-tau217 (pg/mL) | -0.049 (-0.218 to 0.124) | 0.57 | 0.4 |
| **p-tau181** | Roche NeuroToolKit plasma p-tau181 (pg/mL) | -0.069 (-0.234 to 0.098) | 0.42 | REFERENCE |
|  | Quanterix Neurology 4-Plex plasma p-tau181 (pg/ml) | -0.052 (-0.228 to 0.131) | 0.58 | 0.82 |
| **GFAP** | Quanterix Neurology 4-Plex plasma GFAP (pg/mL) | 0.047 (-0.144 to 0.237) | 0.62 | REFERENCE |
|  | Roche NeuroToolKit plasma GFAP (ng/mL) | -0.020 (-0.187 to 0.147) | 0.81 | 0.48 |

**Amyloid PET negative sub-cohort**

| **Analyte** | **Measure** | **Spearman rho** | **p=** | **Comparison** |
| --- | --- | --- | --- | --- |
|  |  | **(95% CI)** |  |  |
| **p-tau217** | C2N Precivity plasma %p-tau217 | 0.114 (-0.013 to 0.238) | 0.08 | REFERENCE |
|  | Janssen LucentAD Quanterix plasma p-tau217 (pg/mL) | 0.055 (-0.080 to 0.186) | 0.41 | 0.43 |
|  | C2N Precivity plasma p-tau217 (pg/mL) | 0.033 (-0.097 to 0.164) | 0.62 | 0.14 |
|  | Fujirebio Lumipulse plasma p-tau217 (pg/mL) | -0.023 (-0.159 to 0.112) | 0.73 | 0.23 |
|  | ALZpath Quanterix plasma p-tau217 (pg/mL) | -0.015 (-0.144 to 0.114) | 0.82 | 0.19 |
| **Aβ42/Aβ40** | C2N Precivity plasma Aβ42/Aβ40 | 0.110 (-0.026 to 0.242) | 0.09 | REFERENCE |
|  | Fujirebio Lumipulse plasma Aβ42/Aβ40 | 0.055 (-0.076 to 0.184) | 0.4 | 0.45 |
|  | Roche NeuroToolKit plasma Aβ42/Aβ40 | -0.022 (-0.150 to 0.110) | 0.73 | 0.26 |
|  | Quanterix Neurology 4-Plex plasma Aβ42/Aβ40 | 0.006 (-0.127 to 0.140) | 0.93 | 0.36 |
| **NfL** | Quanterix Neurology 4-Plex plasma NfL (pg/mL) | 0.074 (-0.064 to 0.214) | 0.28 | REFERENCE |
|  | Roche NeuroToolKit plasma NfL (pg/mL) | 0.072 (-0.064 to 0.204) | 0.27 | 0.37 |
| **p-tau181** | Quanterix Neurology 4-Plex plasma p-tau181 (pg/ml) | -0.034 (-0.168 to 0.102) | 0.63 | REFERENCE |
|  | Roche NeuroToolKit plasma p-tau181 (pg/mL) | 0.031 (-0.098 to 0.162) | 0.64 | 0.92 |
| **GFAP** | Roche NeuroToolKit plasma GFAP (ng/mL) | -0.010 (-0.132 to 0.114) | 0.88 | REFERENCE |
|  | Quanterix Neurology 4-Plex plasma GFAP (pg/mL) | 0.001 (-0.128 to 0.129) | 0.99 | 0.36 |

**Supplementary Table 19. Correlations between rates of change plasma biomarker levels and rates of change in PACC.**Unadjusted Spearman correlations are shown. Correlations between the top performing measure for each analyte and other measures were compared by bootstrapping.

**Full cohort**

| **Analyte** | **Measure** | **Spearman rho** | **p=** | **Comparison** |
| --- | --- | --- | --- | --- |
|  |  | **(95% CI)** |  |  |
| **NfL** | Quanterix Neurology 4-Plex plasma NfL (pg/mL) | -0.271 (-0.372 to -0.163) | <0.01 | REFERENCE |
|  | Roche NeuroToolKit plasma NfL (pg/mL) | -0.230 (-0.328 to -0.125) | <0.01 | 0.18 |
| **p-tau217** | Fujirebio Lumipulse plasma p-tau217 (pg/mL) | -0.207 (-0.311 to -0.098) | <0.01 | REFERENCE |
|  | C2N Precivity plasma p-tau217 (pg/mL) | -0.176 (-0.282 to -0.068) | <0.01 | 0.53 |
|  | Janssen LucentAD Quanterix plasma p-tau217 (pg/mL) | -0.168 (-0.273 to -0.062) | <0.01 | 0.37 |
|  | ALZpath Quanterix plasma p-tau217 (pg/mL) | -0.150 (-0.258 to -0.040) | <0.01 | 0.18 |
|  | C2N Precivity plasma %p-tau217 | -0.100 (-0.206 to 0.004) | 0.05 | 0.09 |
| **GFAP** | Quanterix Neurology 4-Plex plasma GFAP (pg/mL) | -0.170 (-0.280 to -0.053) | <0.01 | REFERENCE |
|  | Roche NeuroToolKit plasma GFAP (ng/mL) | -0.102 (-0.208 to 0.007) | 0.05 | 0.03 |
| **p-tau181** | Quanterix Neurology 4-Plex plasma p-tau181 (pg/ml) | -0.107 (-0.212 to 0.001) | 0.06 | REFERENCE |
|  | Roche NeuroToolKit plasma p-tau181 (pg/mL) | -0.089 (-0.196 to 0.022) | 0.09 | 0.93 |
| **Aβ42/Aβ40** | Quanterix Neurology 4-Plex plasma Aβ42/Aβ40 | -0.098 (-0.206 to 0.008) | 0.08 | REFERENCE |
|  | Fujirebio Lumipulse plasma Aβ42/Aβ40 | -0.096 (-0.199 to 0.008) | 0.06 | 0.91 |
|  | Roche NeuroToolKit plasma Aβ42/Aβ40 | -0.059 (-0.161 to 0.043) | 0.26 | 0.53 |
|  | C2N Precivity plasma Aβ42/Aβ40 | -0.014 (-0.115 to 0.086) | 0.79 | 0.32 |

**Amyloid PET positive sub-cohort**

| **Analyte** | **Measure** | **Spearman rho** | **p=** | **Comparison** |
| --- | --- | --- | --- | --- |
|  |  | **(95% CI)** |  |  |
| **Aβ42/Aβ40** | Fujirebio Lumipulse plasma Aβ42/Aβ40 | -0.257 (-0.415 to -0.085) | <0.01 | REFERENCE |
|  | Quanterix Neurology 4-Plex plasma Aβ42/Aβ40 | -0.146 (-0.321 to 0.037) | 0.12 | 0.26 |
|  | Roche NeuroToolKit plasma Aβ42/Aβ40 | -0.106 (-0.283 to 0.079) | 0.22 | 0.07 |
|  | C2N Precivity plasma Aβ42/Aβ40 | 0.029 (-0.150 to 0.203) | 0.73 | 0.03 |
| **NfL** | Quanterix Neurology 4-Plex plasma NfL (pg/mL) | -0.222 (-0.390 to -0.039) | 0.02 | REFERENCE |
|  | Roche NeuroToolKit plasma NfL (pg/mL) | -0.214 (-0.370 to -0.046) | 0.01 | 0.80 |
| **p-tau217** | Fujirebio Lumipulse plasma p-tau217 (pg/mL) | -0.201 (-0.354 to -0.034) | 0.02 | REFERENCE |
|  | C2N Precivity plasma p-tau217 (pg/mL) | -0.151 (-0.319 to 0.032) | 0.07 | 0.40 |
|  | Janssen LucentAD Quanterix plasma p-tau217 (pg/mL) | -0.135 (-0.295 to 0.034) | 0.11 | 0.27 |
|  | C2N Precivity plasma %p-tau217 | -0.086 (-0.258 to 0.092) | 0.31 | 0.19 |
|  | ALZpath Quanterix plasma p-tau217 (pg/mL) | -0.045 (-0.207 to 0.123) | 0.59 | 0.05 |
| **GFAP** | Quanterix Neurology 4-Plex plasma GFAP (pg/mL) | -0.129 (-0.312 to 0.063) | 0.17 | REFERENCE |
|  | Roche NeuroToolKit plasma GFAP (ng/mL) | -0.052 (-0.219 to 0.119) | 0.54 | 0.22 |
| **p-tau181** | Roche NeuroToolKit plasma p-tau181 (pg/mL) | 0.065 (-0.108 to 0.231) | 0.45 | REFERENCE |
|  | Quanterix Neurology 4-Plex plasma p-tau181 (pg/ml) | 0.056 (-0.125 to 0.231) | 0.55 | 0.86 |

**Amyloid PET negative sub-cohort**

| **Analyte** | **Measure** | **Spearman rho** | **p=** | **Comparison** |
| --- | --- | --- | --- | --- |
|  |  | **(95% CI)** |  |  |
| **NfL** | Quanterix Neurology 4-Plex plasma NfL (pg/mL) | -0.198 (-0.323 to -0.065) | <0.01 | REFERENCE |
|  | Roche NeuroToolKit plasma NfL (pg/mL) | -0.149 (-0.274 to -0.020) | 0.02 | 0.16 |
| **p-tau217** | ALZpath Quanterix plasma p-tau217 (pg/mL) | -0.150 (-0.273 to -0.019) | 0.02 | REFERENCE |
|  | C2N Precivity plasma p-tau217 (pg/mL) | -0.149 (-0.269 to -0.022) | 0.02 | 1 |
|  | Fujirebio Lumipulse plasma p-tau217 (pg/mL) | -0.120 (-0.253 to 0.016) | 0.07 | 0.59 |
|  | C2N Precivity plasma %p-tau217 | -0.113 (-0.243 to 0.016) | 0.09 | 0.66 |
|  | Janssen LucentAD Quanterix plasma p-tau217 (pg/mL) | -0.112 (-0.244 to 0.019) | 0.09 | 0.44 |
| **GFAP** | Quanterix Neurology 4-Plex plasma GFAP (pg/mL) | -0.149 (-0.283 to -0.012) | 0.03 | REFERENCE |
|  | Roche NeuroToolKit plasma GFAP (ng/mL) | -0.112 (-0.244 to 0.026) | 0.09 | 0.33 |
| **p-tau181** | Roche NeuroToolKit plasma p-tau181 (pg/mL) | -0.122 (-0.250 to 0.014) | 0.07 | REFERENCE |
|  | Quanterix Neurology 4-Plex plasma p-tau181 (pg/ml) | -0.083 (-0.217 to 0.048) | 0.23 | 0.71 |
| **Aβ42/Aβ40** | Fujirebio Lumipulse plasma Aβ42/Aβ40 | 0.074 (-0.059 to 0.206) | 0.26 | REFERENCE |
|  | Roche NeuroToolKit plasma Aβ42/Aβ40 | 0.042 (-0.083 to 0.164) | 0.53 | 0.57 |
|  | C2N Precivity plasma Aβ42/Aβ40 | 0.024 (-0.103 to 0.152) | 0.72 | 0.43 |
|  | Quanterix Neurology 4-Plex plasma Aβ42/Aβ40 | -0.003 (-0.134 to 0.132) | 0.96 | 0.39 |
